# When Fairness Is Not Enough: The Disproportionate Contributions of the Poor in a Collective Action Problem

**DOI:** 10.1037/xge0001455

**Published:** 2023-07-20

**Authors:** Eugene Malthouse, Charlie Pilgrim, Daniel Sgroi, Thomas T. Hills

**Affiliations:** 1 Department of Psychology, University of Warwick; 2 Mathematics for Real-World Systems Centre for Doctoral Training, University of Warwick; 3 Department of Economics, University of Warwick

**Keywords:** public goods, collective action, cooperation, meritocracy

## Abstract

Many of our most pressing challenges, from combating climate change to dealing with pandemics, are collective action problems: situations in which individual and collective interests conflict with each other. In such situations, people face a dilemma about making individually costly but collectively beneficial contributions to the common good. Understanding which factors influence people’s willingness to make these contributions is vital for the design of policies and institutions that support the attainment of collective goals. In this study, we investigate how inequalities, and different causes of inequalities, impact individual-level behavior and group-level outcomes. First, we find that what people judged to be fair was not enough to solve the collective action problem: if they acted according to what they thought was fair, they would collectively fail. Second, the level of wealth (rich vs. poor) altered what was judged to be a fair contribution to the public good more than the cause of wealth (merit vs. luck vs. uncertain). Contributions during the game reflected these fairness judgments, with poorer individuals consistently contributing a higher proportion of their wealth than richer participants, which further increased inequality—particularly in successful groups. Finally, the cause of one’s wealth was largely irrelevant, mattering most only when it was uncertain, as opposed to resulting from merit or luck. We discuss implications for policymakers and international climate change negotiations.

## Public Significance Statement

Thinking about many of our current challenges in society as collective action problems, including climate change and pandemics, can help us to better understand how to solve them. This approach highlights the tension that often exists in these situations between individual and collective interests and sheds light on the factors that make people more likely to put collective interests first—which tends to be critical for group success. The factors that we investigate in this study are wealth inequalities between individuals caused by differences in merit or luck. Prior research has shown that these factors are important because they can influence people’s beliefs about what is fair. We find that what people think is fair is not enough to solve a collective action problem and that it tends to be poorer individuals who end up contributing substantially more than their fair share to help their groups succeed.

Humans are a highly cooperative species (Bowles & Gintis, 2011; Henrich & Henrich, 2007). Across the world, people engage in collective action with others every day (Nowak & Highfield, 2011). Sometimes just a handful of individuals are involved, for example, when a group of researchers come together to conduct a study. Other times many millions of individuals are involved, for example, when citizens vote or work together to reduce global warming.

Regardless of how many individuals are involved, human cooperation is vulnerable to free riding (Olson, 1965). This term describes the temptation for each person to “free himself of the trouble and expense, and…lay the whole burden on others” (Hume, 1740, p. 590). Just as a researcher might be tempted to leave a tedious task to their coauthors, organizations might be tempted to avoid taking the costly actions required to reduce their greenhouse gas emissions. The problem is that if every individual involved succumbs to this temptation, the group will inevitably fail to achieve its collective goal—an outcome known as a “tragedy of the commons” (Hardin, 1968).

There are countless factors that may influence one’s temptation to free ride, but one is particularly important and relevant to this study: the behavior of others (Kopelman et al., 2002; Ledyard, 1995; Ostrom, 2008, 2010; Stroebe & Frey, 1982). Much research has shown that a significant proportion of people generally act as conditional cooperators—contributing while others contribute and free riding when others free ride (Croson et al., 2005; Fischbacher & Gächter, 2010; Fischbacher et al., 2001; Gächter, 2006; Kelley & Stahelski, 1970; Keser & van Winden, 2000; Sugden, 1984).

Cooperating on the condition that others are cooperating requires us to judge what constitutes a fair contribution to the joint effort (Elster, 2006; Fehr & Schmidt, 1999; Rabin, 1993; Van Segbroeck et al., 2012). When contributions are financial, two factors have been proposed as important. The first is a person’s total wealth, because people intuitively judge financial contributions in proportional rather than absolute terms (Laming, 1984; Stewart et al., 2005, 2006). In the United Kingdom, for example, *The Sunday Times* Giving List ranks donors according to the proportion of their total wealth donated to charity. This is why the footballer Marcus Rashford topped the list in 2021, despite giving less than a tenth of the sum donated by the runner-up Lord Sainsbury (£229 m; *The Sunday Times*, 2021).

The second factor proposed to be important is the cause of one’s wealth. In recent years, many researchers (e.g., Alan & Ertac, 2017; A. Alesina & Glaeser, 2004; A. Alesina et al., 2018; A. F. Alesina et al., 2001; Fong, 2001; Frank, 2016; Konow, 2000; Koo et al., 2022; Markovits, 2019; Piketty, 1995; Piketty, 2020; Sandel, 2020) have noted the importance of luck and merit in society. They have generally shown that belief in luck rather than merit as the primary determinant of life outcomes generally correlates with stronger individual preferences and public policies in favor of wealth redistribution. One explanation for this is that people are generally more accepting of inequalities arising from merit than from good fortune alone (Adams, 1965; Starmans et al., 2017; Walster et al., 1976)—particularly in Western capitalist cultures (Gonzalez et al., 2022; Son Hing et al., 2011). According to this logic, many people might think it fair for lottery winners to contribute more to the common good than self-made millionaires. But it is unclear whether this logic generally holds and influences behavior in collective action problems.

In reality, wealth is almost always determined by an incalculable combination of luck and merit. Yet there is no consensus in the literature about the impact of this uncertainty on people’s preferences for wealth redistribution. On the one hand, uncertainty regarding the cause of wealth inequalities has been shown to generate an “egalitarian pull” on people with meritocratic preferences (Cappelen et al., 2022). One explanation for this is that, unlike luck and merit, uncertainty does not provide an easily justifiable reason to deviate from equality (Samuelson & Allison, 1994). In other words, luck and merit may be seen as legitimate causes of wealth inequality, whereas an uncertain mixture of luck and merit is not—and people in such a world may seek to redistribute wealth more evenly. On the other hand, uncertainty may bring out self-serving interpretations of wealth whereby people attribute successes to their own efforts and failures to external forces (Miller & Ross, 1975). If this is the case, richer and poorer individuals may become more and less accepting of wealth inequalities, respectively.

In the present study, we empirically investigated the impact of different levels and causes of wealth inequality on beliefs about fairness, individual-level contributions, and group-level outcomes in a collective action problem. We did this by adapting a public good game known as the “collective-risk social dilemma” (introduced by Milinski et al., 2008) in two ways. First, we introduced different levels of wealth by randomly assigning participants to groups of four made up of two richer participants who received an endowment of £20 and two poorer participants who received an endowment of £10. Second, we introduced different causes of wealth by randomly assigning groups to one of three treatment conditions: One in which participants’ endowments were caused by merit (the *merit* treatment); one in which they were caused by luck (the *luck* treatment); and one in which they were caused either by merit or luck, but participants did not know which (the *uncertain* treatment). Below, we explain our adaptation of the collective-risk social dilemma in more detail, as well as our main research questions and hypotheses (Table 1).[Table tbl1]

## The Collective-Risk Social Dilemma

**Figure 1 fig1:**
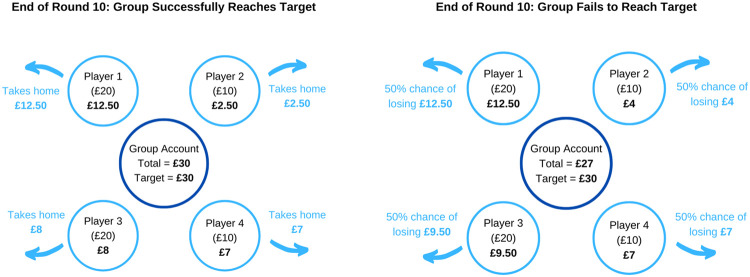
Group Outcomes and Player Payoffs *Note.* See the online article for the color version of this figure.

The collective-risk social dilemma is a specific type of public good game. Public good games have been used for decades to investigate behavior in situations where individual goals conflict with group goals (e.g., Camerer, 2003; Ledyard, 1995; Rapoport, 1988; Sandler, 1992; Van Lange et al., 2013). In brief, participants are given an initial endowment and must decide how much of this to contribute toward a group target in 10 successive rounds. If together they achieve the target sum within the 10 rounds, all players take home the remainder of their endowment (i.e., all funds not contributed to the group account). If the group fails to achieve its target, players face the prospect of losing their remaining endowment. This collective risk creates a social dilemma: the more an individual contributes to the target, the more likely her group isecognicceed, but the less she stands to take home at the end of the game (see Figure 1). This game format is typically described as a threshold public good game, as the group either succeeds (by meeting the threshold) or fails (by falling short). It was designed to represent similar real-world collective action problems such as climate change, which can be understood in terms of threshold dynamics in the sense that we either succeed in limiting global warming to 1.5 °C above preindustrial levels or fail (United Nations, 2015). With the collective-risk social dilemma, we asked the following three research questions:

1 Is what people perceive as fair sufficient to solve collective action problems?
2 How does what people perceive as fair, and how much they are willing to contribute to a public good, depend on the level and cause of their wealth?

[Fig fig1]

Before we detail the experiment we used to answer these questions, we first introduce what is known from prior research and our hypotheses associated with each question.

This figure illustrates how each participant’s final payoff depends on both their individual contribution decisions during the game and their group’s outcome. Each group is made up of two richer participants (Players 1 and 3 here) starting the game with £20 and two poorer participants (Players 2 and 4 here) starting with £10. Players can contribute either £0, £0.75, or £1.50 in each round. If the group succeeds in achieving its target sum of £30 within 10 rounds, players take home what is left of their initial endowment. If the group fails to achieve this target sum, all players face a 50% chance of losing their remaining funds.

## Is What People Perceive as Fair Sufficient to Solve Collective Action Problems?

In many real-world collective action problems, what is judged to be fair may be insufficient to achieve a collective goal. For example, since the Paris Agreement (United Nations, 2015), countries have outlined how they intend to contribute to the reduction of global emissions via nationally determined contributions, which are based partly on what they judge to be fair (Davide et al., 2017). However, according to a recent report from the United Nations Framework Convention on Climate Change (2021), if all 193 governments fulfilled their nationally determined contribution targets, then global greenhouse gas emissions would actually *increase* by 13.7% by 2030—falling far short of the estimated 45% reduction required to limit global warming to 1.5 °C. Another example of fairness not being enough can be found in the European Union Common Fisheries Policy: Between 2001 and 2015, the European Council set national quotas that exceeded scientific advice regarding sustainability by an average of 20% per year (Carpenter et al., 2016).

We anticipated that participants in our experiment might similarly struggle to recognize that what is fair might not be enough (Hypothesis [H1]). In other words, we predicted that participants’ judgments about fair contributions would be insufficient to solve the collective action problem.

## How Does What People Perceive as Fair, and How Much They Are Willing to Contribute to a Public Good, Depend on the Level and Cause of One’s Wealth?

One reason why fairness judgments may be insufficient relates to people’s tendency to hold self-serving beliefs about what is fair (e.g., Babcock & Loewenstein, 1997; Baumeister, 1982; Bernard et al., 2014; Diekmann et al., 1997; Hine & Gifford, 1996; Joireman et al., 1994; Wade-Benzoni et al., 1996). We, therefore, anticipated that richer participants would be more likely than poorer participants to judge it fair that richer players contribute a lower proportion of their wealth than poorer players. And, conversely, poorer participants would be more likely than richer participants to judge it fair that poorer players contribute a lower proportion of their wealth than richer players (Hypothesis [H2]).

In turn, we expected participants’ contributions toward the group target within the game to reflect these self-serving fairness judgments. Based on prior research (e.g., Chan et al., 1996; De Cremer, 2007; Hargreaves Heap et al., 2016; Martinangeli & Martinsson, 2020; Marwell & Ames, 1979; Tavoni et al., 2011; Van Dijk & Wilke, 1993, 1994; Van Lange et al., 2013; Vasconcelos et al., 2014; Vicens et al., 2018), we predicted that richer participants would contribute more than poorer participants in absolute terms; but less in proportional terms (Hypothesis [H3]).

Our remaining hypotheses relate to the effect of different causes of wealth (merit vs. luck vs. uncertainty). Based on the research discussed above, we anticipate two possibilities: (a) merit is seen as the primary indicator of deserved wealth; or (b) both merit and luck (but not uncertainty) are seen as justifiable criteria for wealth inequalities. We believed that (a) was more likely, and therefore predicted that poorer participants in the merit treatment (vs. those in the luck and uncertain treatments) would be expected to contribute a higher proportion of their wealth (Hypothesis [H4]).

In turn, we anticipated that participants’ actual contributions toward the group target during the game would reflect these expectations—with the deserving rich contributing a lower proportion of their wealth than the uncertain and lucky rich (Hypothesis [H5]).

Lastly, we anticipated that these predicted differences in contributions between treatments would have a knock-on effect on group outcomes. We, therefore, predicted that groups in our luck treatment would achieve the target of £30 with a higher success rate than groups in the uncertain and merit treatments (Hypothesis [H6]).

Together, these made up our main hypotheses—all of which are summarized in Table 2 (H3, H4, H5, and H6 were formally preregistered, available at https://osf.io/4expt). If our prediction that fairness judgments would be insufficient turned out to be accurate, participants would not contribute enough to achieve the group target. In the next section, therefore, we identify certain factors that might explain the difference between group success and failure (Table 2).[Table tbl2]

## If What People Perceive as Fair Is Insufficient to Solve the Problem, Under What Conditions Do Groups Still Manage to Succeed?

The ability of groups to succeed despite insufficient views of fairness (H1) will depend on whether certain individuals step up to fill the gap between what is fair and what is required for success. To investigate whether richer or poorer participants stepped up in this way to help their groups succeed, we compared their contributions in successful and unsuccessful groups. If it was the latter then wealth inequalities within groups would increase—particularly within groups that were successful. It remains unclear how richer and poorer participants in luck-based, merit-based, and uncertain groups might respond to such a development because, to our knowledge, the intersection of cause of wealth and wealth inequality has not been examined in a collective risk game.

We also identified two other factors that might help to explain group success: participants’ contributions in the first round and their response to their group not contributing at the rate required to achieve the target (£3 per round). We explain how we intend to test these, along with all of our other hypotheses, in the following section.

## Method

### Participants

We sourced a total of 240 participants via Prolific Academic and Mechanical Turk. We arrived at this sample size via power calculations based on effect sizes detected in previous similar studies (see rationale in our preregistration: https://osf.io/4expt). We collected data initially from 124 participants in April 2021 and (after peer review) from an additional 116 participants in May 2022. We generally collected data from four groups at a time, depending on participant availability, by publishing the study online and accepting participation on a first-come-first-served basis. We originally planned to recruit participants roughly evenly from Prolific and MTurk to avoid any biases associated with either pool (Litman et al., 2021; Palan & Schitter, 2018; Paolacci et al., 2010). However, grouping people up was much more straightforward on Prolific due to greater participant availability, and so our final sample consisted of 188 participants from Prolific and 52 participants from MTurk. All results reported below reflect pooled Prolific Academic and Mechanical Turk data; in the online supplemental materials, we separate results from these two sample populations and note differences between the two (see Figure S3 in the online supplemental materials).

Regardless of platform, all participants were over the age of 18 and entered their age range and gender at the start of the experiment: 34% were aged 18–24; 43% were aged 25–34; 14% were aged 35–44; 7% were aged 45–54; and 3% were aged 55+; while 44% of all participants were female, 55% were male, and 1% identified as nonbinary. Participants received pro rata payment of £7.50 per hour, as recommended by Prolific Academic. In addition, they had the opportunity to earn a bonus payment depending on the outcome of the experiment (*M* = £6.69, *SD* = £4.70). This rate (and the whole experiment) was approved by the University of Warwick’s Psychology Department Research Ethics Committee. The experiment was programmed using oTree, a platform that enables researchers to build and run online experiments (Chen et al., 2016).

### Experiment Design

After participants read an information sheet and consented to the terms of the experiment (see supplemental materials) they were randomly assigned to groups of four, which in turn were randomly assigned to the merit, luck, or uncertain treatments. Every group was made up of two richer participants, who started the game with an endowment of £20, and two poorer participants who started the game with £10. The level of inequality was therefore identical in each group, but the cause of these inequalities differed between our three treatments:

• In the merit treatment, participants’ endowments were determined by their performance in the effort task. In each group, the two highest-scoring participants received £20 to start the game and the two lowest-scoring participants received just £10. All participants were explicitly informed about this meritocratic allocation both before and after they completed the task.
• In the luck treatment participants’ endowments were determined randomly by a lottery. This meant that their effort task performance had no bearing on whether they started the game with £20 or £10, and we told participants that this was the case both before and after they completed the task. To incentivize completion of the task, we gave a £1 bonus payment to the highest-scoring member of each group at the very end of the experiment.
• In the uncertain treatment, participants did not know the true determinant of their wealth. In each group, two randomly chosen participants’ endowments were determined by their performance in the effort task, with the higher scoring of these two receiving £20 and the lower-scoring player receiving £10. For the other two group members, endowments were determined by a lottery, with the winner receiving a £20 endowment and the loser £10. Participants in these groups were told that their endowment was determined either by their task performance or by the lottery—but they were not told which.

The structure of the experiment was exactly the same for every participant. It began with an effort task previously used by Niederle and Vesterlund (2007) and Oswald et al. (2015). This entailed adding up sequences of five random two-digit numbers for 5 min (i.e., 16 + 82 + 51 + 55 + 26 = ?) with participants receiving one point per correct answer. While we asked participants to refrain from using a calculator, we recognized that this request was unlikely to be followed in an online environment. However, as stated in our preregistration, this was not a primary concern because we ultimately wanted participants to believe that their degree of effort was correlated with their rewards. This applies if some people are better at mental arithmetic than others and even if some people are using a calculator (since using a calculator for five minutes still represents an effortful activity).

Once all participants in a group had completed this task, we told them whether they would start the game with £20 or £10 and confirmed whether this was determined by merit, luck, or one of the two (depending on their treatment). We then explained the rules of the collective-risk social dilemma game using illustrations similar to Figure 1. We made it clear what would happen if the group succeeded (all players would retain all funds not contributed to the group account) and if it failed (all players would face a 50% chance of losing all funds not contributed). We then tested their understanding with three comprehension questions, the first of which asked how much each player would have to contribute on average for the group to achieve its target (£7.50). We then asked them what, in their opinion, they considered to be a fair total contribution toward the group target from richer and poorer players (see the online supplemental materials for full pregame questionnaire). Participants had to answer the comprehension questions correctly before they could proceed to the game, which helped to ensure that their responses to the fairness questions reflected their opinions rather than their understanding of the game. The first round of the game began after every participant in the group had completed these steps.

The collective-risk social dilemma was played over 10 rounds. At the start of each round, participants were asked how much of their endowment (£0/£0.75/£1.50) they would like to contribute toward the group target of £30. We gave participants three contribution options, following Milinski et al. (2008), mainly because it enabled participants to quickly estimate what others had contributed at the end of each round. We set the target at £30 because group success would require each group member to contribute half of their wealth on average. And we set the probability of losing the remainder of one’s endowment in the event of group failure at 50% because it meant that players in each group faced the same expected earnings whether they chose to free ride (i.e., contribute nothing to a failing group) or all cooperate (i.e., give half of their endowment). As an example, a poorer participant would, in expectation, stand to take home £10 × 50% = £5 by free riding and £10 − £5 = £5 by contributing 50% of their wealth, providing others in the group did the same. At the start of each round, we showed participants the round number, how much remained of their endowment, how much the group had contributed in total so far, and how much more the group needed to contribute to achieve its target. At the end of the game, participants were told their group outcome and, in the event of failure, whether or not they had survived the collective risk and therefore retained the remainder of their endowment.

In summary, we created groups made up of two richer and two poorer participants and manipulated the cause of these wealth inequalities (merit vs. luck vs. uncertain). In 10 successive rounds, participants decided how much to contribute to the group target. This design enabled us to test our hypotheses regarding fairness judgments, contributions, and group outcomes. We describe our statistical tests, which were carried out in R and JASP (JASP Team, 2020), in the next section.

### Main Hypotheses and Statistical Tests

*Hypothesis 1 (H1):* Fairness will be insufficient: participants will on average judge it fair that richer and poorer players contribute less than 50% of their wealth

To test H1, we investigated whether participants’ judgments about fair total contributions were on average less than 50% of richer and poorer players’ wealth (the level required to achieve the group target). As a secondary measure, we calculated what they judged to be fair for the group as a whole to contribute by summing an individual’s responses to these questions and multiplying this figure by two; and then checking the proportion of participants for whom this total was insufficient (i.e., less than the group target of £30).

*Hypothesis 2 (H2):* Richer (poorer) participants will judge it fair that they contribute a lower proportion of their wealth than poorer (richer) participants

To test H2, we first coded participants’ responses to our fairness questions according to one of three fairness principles (similar to Reindl, 2022):

• Progressive: if they judged that rich players should contribute a higher proportion of their wealth than poor players;
• Equal: if they judged that rich and poor players should contribute the same proportion of their wealth; or
• Regressive: if they judged that poor players should contribute a higher proportion of their wealth than rich players.

We then conducted chi-squared tests to compare the proportion of richer and poorer participants whose responses reflected progressive and regressive principles. H2a was that a higher proportion of richer (poorer) participants’ responses would reflect the regressive (progressive) principle—which would represent a marker for self-serving bias.

We also compared what richer and poorer participants actually judged to be a fair contribution from richer and poorer players. H2b was that richer participants’ response to the question of how much richer players should contribute would on average be lower in proportional terms than the response from poorer participants. H2c was the exact opposite: poorer participants’ response to the question of how much poorer players should contribute would on average be lower in proportional terms than the response from richer participants. We tested H2b and H2c by conducting standard and Bayesian analyses of variance (ANOVAs) to test for wealth effects on these responses.

*Hypothesis 3 (H3):* Richer participants will contribute more than poorer participants in absolute terms, but less in proportional terms

We tested H3 by comparing richer and poorer participants’ mean absolute and mean proportional total contributions from live rounds (i.e., rounds in which the group target had not already been achieved) with standard and Bayesian ANOVAs. In addition, we conducted two linear multilevel models: the first with absolute contributions as the dependent variable and the second with proportional contributions as the dependent variable; and both with fixed wealth effects and random intercepts for rounds, individuals, and groups. We did this to take the hierarchical nature of our data into account (since rounds were nested in individuals and individuals were nested in groups) and did not use random slopes for these factors because they did not significantly improve model fit.

*Hypothesis 4 (H4):* Individuals in the merit treatment will expect the poor to contribute a higher proportion of their wealth

We tested H4 by conducting standard and Bayesian ANOVAs with treatment and wealth as independent variables and participants’ responses to the questions of what would be fair for richer and poorer participants to contribute as the dependent variables. A significant treatment effect would indicate that the cause of wealth influenced participants’ fairness judgments.

*Hypothesis 5 (H5):* The deserving rich will contribute a lower proportion of their wealth than the uncertain and the lucky rich

We tested H5 by conducting standard and Bayesian ANOVAs to compare proportional contributions from richer participants between our treatments; and again, for additional robustness, by running a multilevel model with fixed wealth and treatment effects and random intercepts for rounds, individuals, and groups to take the variance from these factors into account.

*Hypothesis 6 (H6):* Luck-based groups would be more successful than merit-based and uncertain groups

We tested H5 by comparing the proportion of groups that were successful in each treatment using chi-squared tests.

### Exploratory Hypotheses

In our introduction, we also highlighted certain factors that might help to explain the difference between group success and failure. The first was whether richer or poorer participants contributed more than their fair share. To investigate this, we compared their contributions in successful and unsuccessful groups (similar to Tavoni et al., 2011) with standard and Bayesian analyses of covariance that included proportional contributions as the dependent variable, wealth as the independent variable, and group success as a covariate. For additional robustness, we conducted a multilevel model that included proportional contribution as the dependent variable and fixed wealth and group success effects, as well as random round, individual, and group intercepts. This model enabled us to test for an interaction between wealth and group success, which would show whether richer or poorer participants stepped up to help their groups succeed.

If it ended up being poorer participants who stepped up, then wealth inequality within groups would increase—particularly in successful groups. We tested this by calculating and comparing the mean Gini coefficients of successful and unsuccessful groups at the end of the game based on participants’ remaining endowments (before those in unsuccessful groups faced the prospect of losing their funds) with standard and Bayesian ANOVAs.

We discussed two other factors that might be important for group success: (a) participants’ contributions on the first round; and (b) their response to their group contributing less than the required rate of contribution (£3 per round). We tested the first by comparing mean first-round contributions between wealth and treatment levels with standard and Bayesian ANOVAs. We tested the second by calculating the “slack” before each round (defined as the difference between a cumulative contribution of £3 per round and the current group total) and running multilevel models on data from richer and poorer participants with their contributions as the dependent variable, fixed slack and treatment effects, and random round, individual, and group intercepts. A significant Slack × Treatment interaction term would indicate that richer or poorer participants in a certain treatment were responding differently to their peers in other treatments.

### Transparency and Openness

• Citation: all methods developed by others (e.g., oTree, JASP) are appropriately cited in the text and listed in the references section.
• Data and code transparency: anonymized processed data on which study conclusions are based, as well as reproducible computer code used for statistical analyses, are available at https://osf.io/8kn57/.
• Preregistration: the study design, hypotheses, and analysis plan were preregistered and are available at https://osf.io/4expt.
• Materials transparency: examples of the materials described in the methods section are shown in the online supplemental materials.

## Results

### Is What People Perceive as Fair Sufficient to Solve Collective Action Problems?

Our first hypothesis (H1) concerned people’s beliefs about what was fair and whether this was enough to solve the collective action problem. Before analyzing participants’ judgments, we excluded 12 responses above £15 for the question of how much richer players should contribute because this was not practically possible (since the maximum players could contribute in each of the 10 rounds was £1.50). We also excluded four responses above £10 for the question of how much poorer players should contribute (since this exceeded their endowment).

**Figure 2 fig2:**
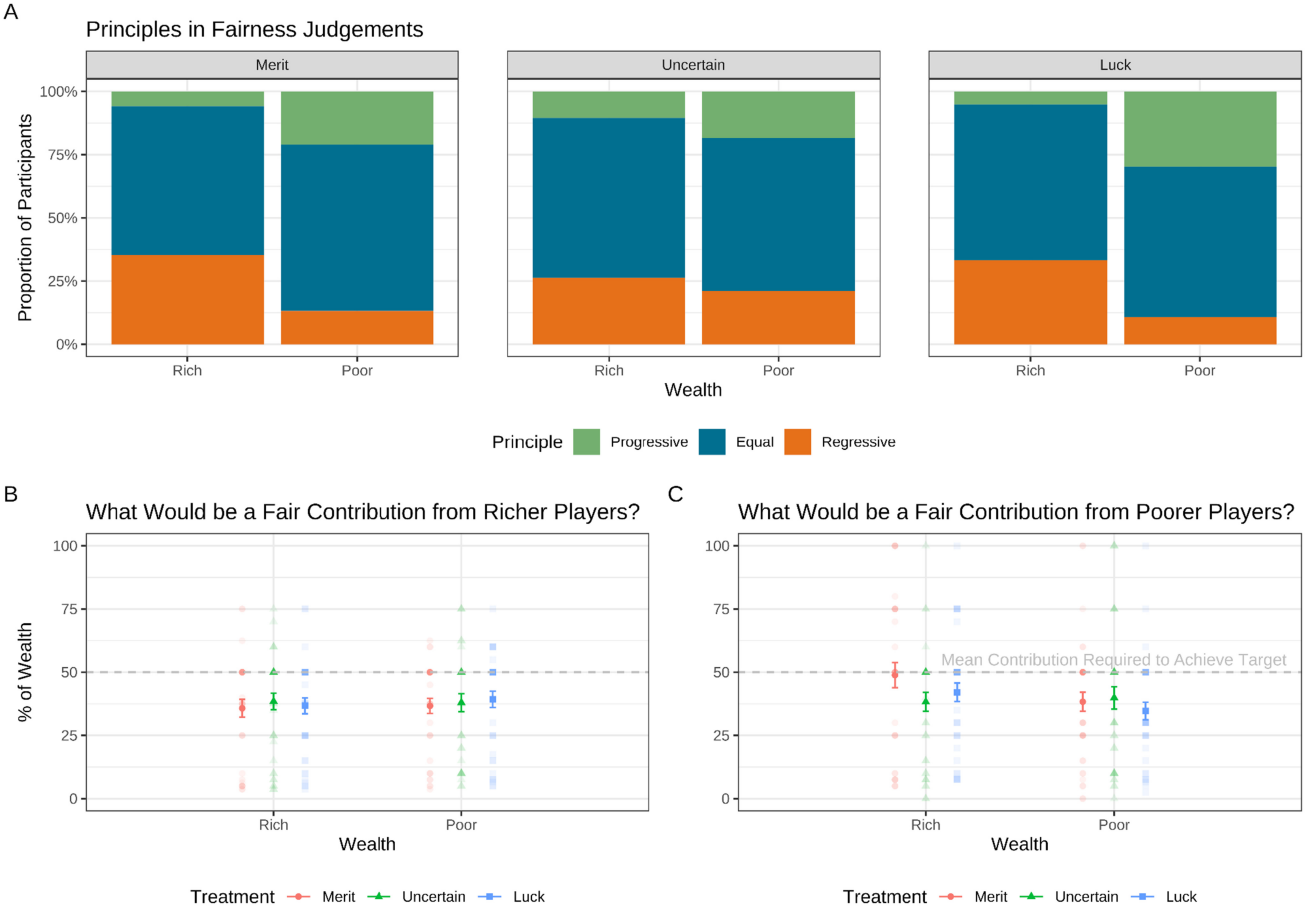
Participants’ Beliefs About Fair Contributions *Note*. See the online article for the color version of this figure.

Across all three treatments, participants on average judged it fair for richer participants to contribute £7.50 (37.5% of their wealth) and for poorer participants to contribute £4.04 (40.4% of their wealth). This was insufficient to solve the collective action problem—which on average required everyone to contribute 50% of their wealth (see Figure 2). Furthermore, what 42.4% of participants judged to be fair for their group to contribute was insufficient, totaling less than £30. This was higher than the proportion of people (7.6%) whose responses added up to more than £30. We found a similar pattern when including all responses: 39.6% of participants judged to be fair an amount that was insufficient, while 13.8% judged to be fair an amount that exceeded £30. On account of this consistent skew below the group threshold and on account of the fact that these fairness judgments were elicited immediately after three comprehension questions (one of which directly asked how much participants needed to contribute on average to succeed, and all of which participants had to answer correctly to proceed to the game), it seems unlikely that these results represent a fundamental misunderstanding of the game. These results, therefore, provide support for H1.[Fig fig2]

### How Does What People Perceive as Fair, and How Much They Are Willing to Contribute to a Public Good, Depend on the Level and Cause of Their Wealth?

H2 related to self-serving bias in fairness judgments from richer and poorer participants. As illustrated in Plot A in Figure 2, we found that after categorizing participants’ judgments according to one of three fairness principles (progressive vs. equal vs. regressive), a significantly higher proportion of poorer participants’ responses (23.0%) were progressive compared to richer participants, 7.2%; χ^2^(1) = 9.67, *p* = .002. And a significantly higher proportion of richer participants’ responses (31.5%) were regressive compared to poorer participants, 15.0%; χ^2^(1) = 7.64, *p* = .006. However, as illustrated in Plots B and C in Figure 2, we did not detect a significant wealth effect on participants’ responses in proportional terms to the question of what would be a fair total contribution from richer players, *F*(1, 226) = 0.13, *p* = .719; *BF*_01_ = 6.5, or poorer players, *F*(1, 234) = 2.66, *p* = .104; *BF*_01_ = 2.0. These results provide strong evidence for H2a but no evidence for H2b or H2c—indicating that participants’ level of wealth did influence which fairness principle their judgments reflected, but did not influence their responses significantly.

Plots show participants’ responses to two questions in the pregame questionnaire: “In your opinion, what would be a fair total contribution in £ to the group account during the game?” from players starting with £20 and players starting with £10. Plot A shows the fairness principles that participants’ responses reflected across wealth and treatment levels—Progressive meant they believed poorer players should contribute a higher proportion of their wealth than richer players; Regressive meant the opposite; and Equal meant they believed players should contribute equal proportions. Plots B and C illustrate actual responses and have been converted into proportional terms as a percentage of each type of player’s wealth. Plot B shows that richer players were on average expected to contribute 37.5% of their wealth, while Plot C shows poorer players were expected to contribute 40.4% of theirs. In these plots, points in the background represent raw data and are slightly transparent to show overlapping responses; summary points show mean responses from richer and poorer participants across treatment conditions with bars representing the standard errors. The dashed gray line represents the average level of contribution required (50%) for groups to achieve their target.

H3 was that contributions would reflect fairness judgments: Richer participants would contribute more to the group account than poorer participants in absolute terms, but less in proportional terms. Standard and Bayesian ANOVAs indicated that in absolute terms, richer participants on average contributed more than poorer participants, *F*(1, 238) = 89.25, *p* < .001; *BF*_10_ = 1.257e + 15. In proportional terms, however, poorer participants contributed a higher proportion of their wealth (*M* = 62.3%) than richer participants, *M* = 47.6%, *F*(1, 238) = 26.52, *p* < .001, *BF*_10_ = 23,862. Our multilevel models similarly indicated that poorer participants contributed less in absolute terms, *t*(238) = −10.3, *p* < .001, but more in proportional terms, *t*(238) = 5.4, *p* < .001. These results supported H3 and also showed that both richer and poorer participants contributed a higher proportion of their wealth than was judged by all to be fair (rich: 47.6% vs. 37.5%; poor: 62.3% vs. 40.4%)—with poorer participants doing so to a greater extent.

Our next set of hypotheses was related to the effect of different causes of wealth on fairness judgments (H4); contributions toward the group target (H5); and group success (H6). For H4, we did not detect a treatment effect on fairness judgments (see Plots B and C in Figure 2). This was equally true for the question of what would be fair for richer participants to contribute, *F*(2, 222) = 0.22, *p* = .803; *BF*_01_ = 17.9), as it was for the question of what it would be fair for poorer participants to contribute, *F*(2, 230) = 0.98, *p* = .378; *BF*_01_ = 9.9. In other words, fairness judgments were not influenced by whether inequalities had been determined by merit, luck, or one of the two.

**Figure 3 fig3:**
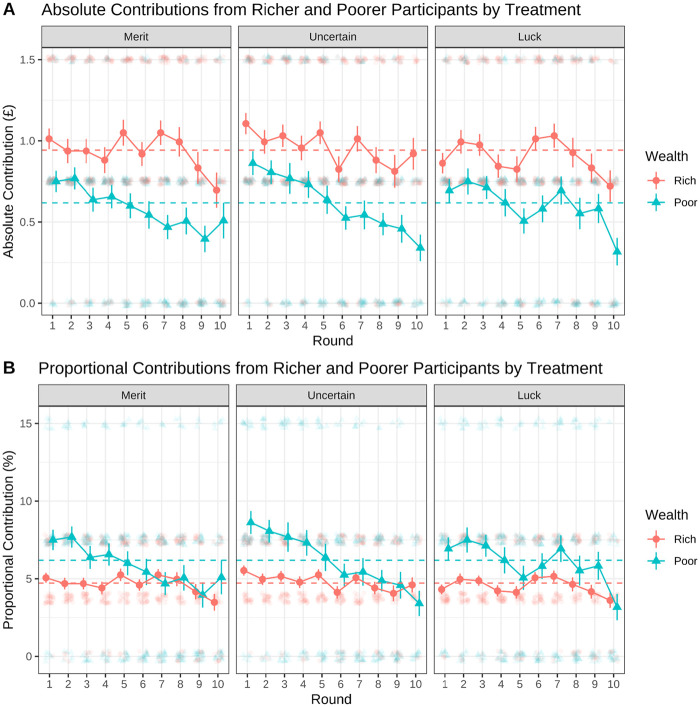
Participant Contributions in Absolute and Proportional Terms *Note*. See the online article for the color version of this figure.

Similarly, we did not find that the cause of wealth influenced participants’ actual contributions during the game (H5). As shown in Figure 3, richer and poorer participants’ absolute contributions were similar across the merit, luck, and uncertain treatments—results of standard and Bayesian ANOVAs: (rich) *F*(2, 117) = 0.32, *p* = .726; *BF*_01_ = 9.7; (poor) *F*(2, 117) = 3.55, *p* = .702; *BF*_01_ = 9.4. In fact, the outputs of both Bayesian ANOVAs indicated that these differences were around 10 times more likely to be explained by the null hypothesis. Equally, our multilevel model did not detect a significant treatment effect (see Table S1 in the online supplemental materials for full model output and Figure S5 in the online supplemental materials for model predictions across treatment and wealth levels). These results provide strong evidence that the cause of wealth was largely irrelevant to the poor contributing substantially more than their fair share.[Fig fig3]

**Figure 4 fig4:**
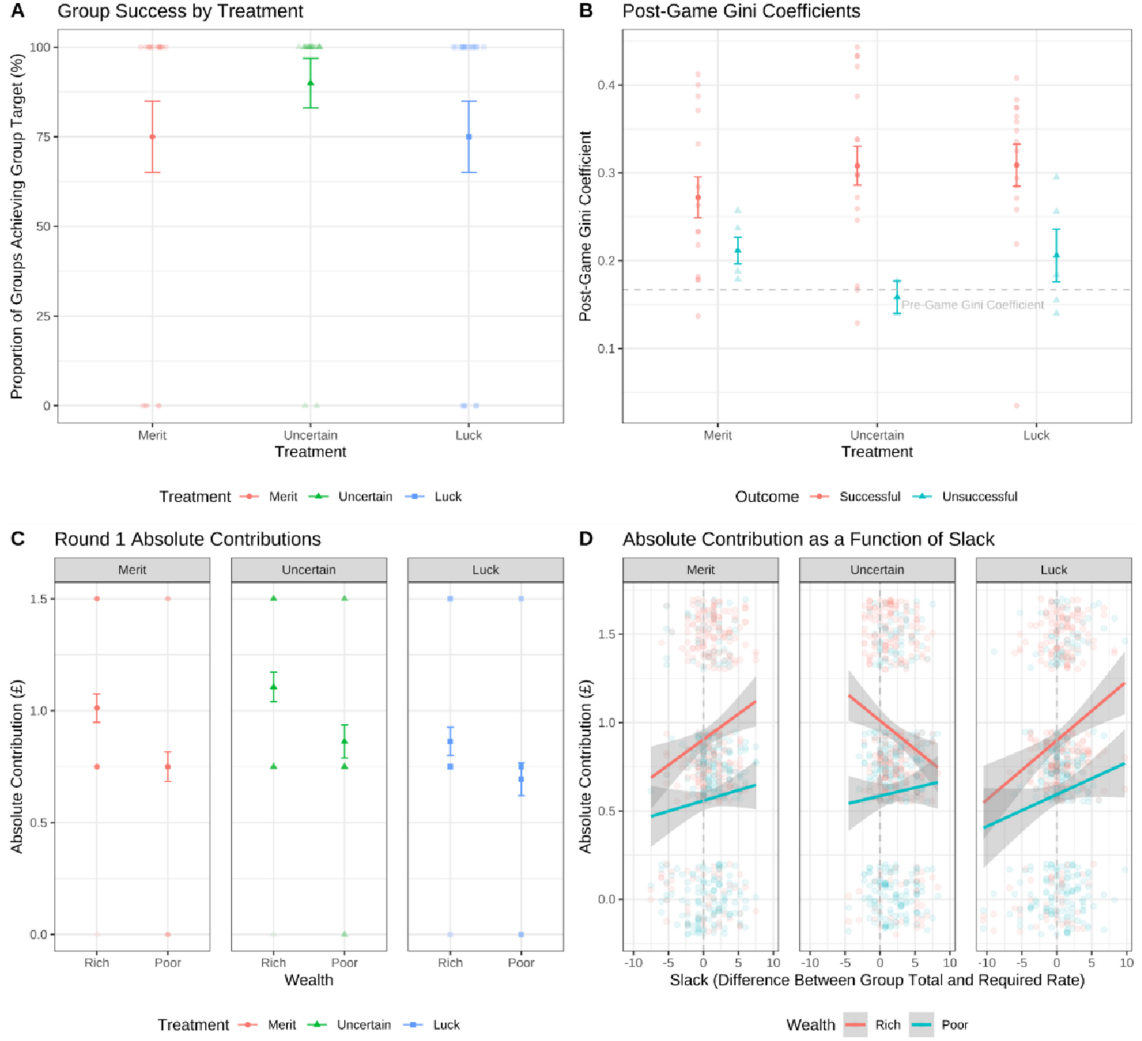
Group Outcomes, Group Inequality, Cumulative Contributions, and Slack *Note*. See the online article for the color version of this figure.

In line with this finding, we did not find that different causes of wealth resulted in significantly different outcomes at the group level (H6). While uncertain groups had a success rate of 90%, merit-based and luck-based groups achieved the target 75% of the time, χ^2^(2) = 1.88, *p* = .392, *BF*_01_ = 17.4 (see Plot A in Figure 4).[Fig fig4]

Plots show the mean contributions (excluding rounds in which the group target had already been met) of richer and poorer participants by treatment. Points in the background represent raw data: they are faded and jittered to show overlapping responses. Larger colored points represent the mean; bars represent the standard error. Dashed horizontal lines represent the mean total contribution from all richer and poorer participants across all three treatment conditions. Plot A shows the absolute value of contributions (£0/£0.75/£1.50). Plot B shows contributions in proportional terms: with a £1.50 contribution being 7.5% of a richer participant’s and 15% of a poorer participant’s endowment; a £0.75 contribution represented as 7.5% and 3.75%, respectively; and a £0 contribution represented as 0%.

### If What People Perceive as Fair Is Insufficient to Solve the Problem, Under What Conditions Do Groups Still Manage to Succeed?

To answer this question, we first compared richer and poorer participants’ contributions in successful and unsuccessful groups. We found that richer participants contributed 49.4% of their wealth in successful groups and 40.6% of their wealth in unsuccessful groups—a difference of 8.8 percentage points. On the other hand, poorer participants contributed 67.8% of their wealth in successful groups and 40.0% of their wealth in unsuccessful groups—a difference of 27.8 percentage points. Standard and Bayesian analyses of covariance detected wealth, *F*(1, 237) = 29.72, *p* < .001; *BF*_10_ = 23,862, and group success effects, *F*(1, 237) = 29.65, *p* < .001; *BF*_10_ = 23,164; while our multilevel model highlighted a significant interaction of poor wealth and group success, *t*(236) = 3.0, *p* = .003. This interaction indicated that the effect of poor wealth on proportional contributions depended on group success.

These results suggested that poorer participants’ contributions were particularly relevant for group outcomes. We verified this by comparing Gini coefficients within groups at the end of the game (see Plot B in Figure 3), which were higher in successful groups (*M* = 0.30) than unsuccessful groups, *M* = 0.20; *F*(1, 54) = 12.0, *p* = .001; *BF*_10_ = 33.3, and were not moderated by treatment, *F*(1, 54) = 0.22, *p* = .800; *BF*_10_ = 0.007. Together, these findings indicated that poorer participants stepping up to contribute substantially more than their fair share helped to explain group success.

Plot A shows the proportion of groups who were successful, with points in the background representing groups and summary points showing the mean success rates and error bars representing the standard error (where 100 represents group success and 0 represents group failure). Plot B shows the Gini coefficients of successful and unsuccessful groups at the end of the game, illustrating how within-group inequality tended to increase over time—particularly in successful groups. The horizontal dashed gray line represents the Gini coefficient of all groups at the start of the game (0.17); points in the background represent groups; solid colored points and bars represent means and standard errors for successful and unsuccessful groups in each treatment. Plot C shows cumulative group contributions over time in each treatment. Points represent the mean contribution from groups in each treatment in each round, and bars represent the standard error. The diagonal gray line illustrates the required rate of cumulative group contributions to succeed in reaching the target sum of £30 within 10 rounds. Plot D shows contributions from richer and poorer participants in each treatment as a function of the difference between the current group total and the required contribution rate of £3 per round. For example, a group that has collectively contributed £25 after nine rounds would be plotted at −2, because they are £2 behind the curve.

If the poor contributing a greater proportion of their wealth helped to explain group success across all three treatments, what explained the higher success rate of groups in our uncertain treatment? One explanation, illustrated in Plot C in Figure 4, was that participants in this treatment on average contributed more toward the group target in the first round (*M* = £0.98) than participants in the merit (*M* = £0.88) and luck treatments, *M* = £0.78, *F*(2, 237) = 4.39, *p* = .013; *BF*_10_ = 2.17. This meant that uncertain groups for the most part had to sustain rather than build momentum.

A second explanation, illustrated in Plot D in Figure 4, was that the uncertain rich were more likely than the deserving and lucky rich to support their groups when they fell behind the required rate of contribution. We verified this by running separate multilevel models with contributions from richer and poorer participants as dependent variables: the only significant interaction we detected was between the slack variable and the uncertain treatment on richer participants’ contributions, *t*(168) = −2.5, *p* = .015; see Table S2 in the online supplemental materials for full model outputs and Figure S6 in the online supplemental materials for model predictions across treatment and wealth levels. In summary, the uncertain rich picked up the slack when their groups fell behind the required rate of contribution in a way that the deserving and lucky rich did not (Table 3).[Table tbl3]

## Discussion

Our main finding is that what many people perceive to be fair is insufficient to solve the collective action problem at hand. Overall, participants judged it fair that richer participants contribute 37.5% and poorer participants contribute 40.4% of their wealth—both of which fell short of the average 50% figure required to solve the problem. Similarly, what a significant proportion of individuals (42.4%) judged to be fair for their group as a whole to contribute was not enough for group success. This was considerably higher than the proportion of individuals (7.6%) who judged it fair that their group should contribute *more* than the target of £30. This finding supports H1 and is highly relevant to a host of real-world collective action problems, including climate change and sustainable fishing, where what is judged to be fair may ultimately be insufficient.

One explanation for this finding was that fairness judgments were often self-serving. This was evident in the fact that 23% of poorer participants (vs. just 7.2% of richer participants) judged progressive wealth redistribution to be fair; while 31.5% of richer participants (vs. just 15% of poorer participants) judged regressive wealth redistribution to be fair. These self-serving interpretations of fairness, which partially supported H2, have been cited as a major barrier in international climate negotiations (Brick & Visser, 2015; Carlsson et al., 2013; Lange et al., 2010; Reindl, 2022).

Even when fairness judgments are not self-serving, if they are insufficient then group success will require some members to contribute more than what is deemed to be their fair share. In our study, it was predominantly poorer participants who stepped up in this way and who had a disproportionate influence on group outcomes. Despite having less to give, and despite it generally being judged fair that they contribute 40.4% of their endowment, they consistently contributed a higher proportion of their wealth (*M* = 62.3%) than richer participants (*M* = 47.6%). This finding supported H3 and was particularly true in successful groups, in which wealth inequality increased as a result.

The level of people’s wealth therefore had an important effect on fairness judgments and contributions, unlike the cause of their wealth. We did not find evidence to support H4 (that fairness judgments would differ between treatments) or H5 (that contributions would differ between treatments). In other words, richer participants generally contributed a lower proportion of their wealth regardless of its cause. The rich did this despite the fact that they had more to lose in financial terms than poorer participants, which might have motivated them to cooperate more.

Contrary to H6, we found that uncertain (rather than luck-based) groups were the most successful. We attributed this to two factors: uncertain participants’ higher contributions in Round 1 and the response of the uncertain rich to their group contributing less than the required rate of contribution. One possible explanation for these differences, discussed in the introduction, is that uncertainty about the cause of inequality can generate an egalitarian pull on the behavior of meritocrats (Cappelen et al., 2022). The reason for this is that people may view uncertainty as an unfair way of distributing wealth in comparison with merit or luck. In an experiment by Samuelson and Allison (1994), for instance, luck and merit were both viewed as valid causes of inequality. More spurious causes of inequality on the other hand were not. In our experiment, the deserving and lucky rich may have similarly viewed merit and luck as equally valid causes of inequality and been less willing to redistribute wealth as a result—unlike the uncertain rich. It is also possible that conflicting beliefs about the legitimacy of inequality between richer and poorer participants in luck- and merit-based groups may have hampered group coordination. We acknowledge, however, that validating these explanations would require further research.

It is also worth highlighting here what we believe to be the main limitations of our experiment, which relate to the generalizability of our findings. Firstly, it is unlikely that our merit and luck manipulations accurately reflect how people think about these phenomena in relation to their life outcomes in natural settings. In practice, they are often conflated, as illustrated by the Latin proverb, “fortune favors the brave” (Flusfeder, 2022). Secondly, failure in real-world collective action tends to involve higher stakes than simply losing one’s endowment. It is likely that our participants were, therefore, less concerned about collective failure than individuals might be in real-world equivalent situations, which may result in different behavioral responses. Future research could, therefore, further explore attributions of merit and luck in experimental and natural settings, or it could consider increasing the stakes. In addition, it could explore how endowments based on performance shape people’s level of trust in others and their feelings about other members of their group. And how these feelings might, in turn, influence both individual-level contribution behavior and group-level outcomes.

In conclusion, our findings illustrate how what is perceived to be fair in collective action problems may be insufficient for the attainment of group goals. On an individual level, our results highlight the general reluctance of richer individuals to sacrifice personal wealth and reduce inequality to support joint efforts to avert collective risks. Our findings also highlight the disproportionate influence of the poor in these situations, whose willingness to contribute considerably more than what they themselves deemed as fair was crucial to group success. Our results relating to the effect of different causes of wealth suggest that promoting a meritocratic message about the cause of wealth inequalities is unlikely to support cooperation in collective action problems; nor is telling people that they have been either lucky or unlucky (Frank, 2016; Markovits, 2019; Sandel, 2020). Instead, perhaps there is greater promise in highlighting the uncertainty inherent in any attempt to calculate the relative roles of luck and merit in our respective histories.

On a group level, our results illustrate both the value of early contributions and the value of individuals who are willing to pick up the slack if the group is not contributing at the rate required to solve the collective action problem. These were important factors underlying the higher success rate of groups in our uncertain treatment. The implication for policymakers is that if a group falls behind this required rate it may be unwise to expect its members, richer or poorer, to mitigate the impending disaster later down the line.

### Constraints on Generality

Our results do not capture the impact of the high stakes associated with real-world collective action problems such as climate change and pandemics. In addition, our sample was made up of participants available on Prolific Academic and MTurk and consequently reflects the populations signed up to these platforms (see Table S3 in the online supplemental materials for details). Real-world collective action problems often involve individuals from many more countries—many of whom are likely to hold different beliefs about both fairness and the relationship between merit, luck, and inequality. Lastly, the contribution options available to participants in each round (£0/£0.75/£1.50) were artificially restrictive to force individuals to solve the problem over multiple rounds of interaction, similar to many real-world problems; in reality, individuals in collective action problems are likely to have much greater control over their contributions. Beyond these limitations, we have no reason to believe that the results depend on other characteristics of the participants, materials, or context.

## Supplementary Material

10.1037/xge0001455.supp

## Figures and Tables

**Table 1 tbl1:** Participant Types

Wealth	Merit treatment	Luck treatment	Uncertain treatment
Rich (£20)	Deserving rich	Lucky rich	Uncertain rich
Poor (£10)	Deserving poor	Unlucky poor	Uncertain poor
*Note*. The table summarizes the different levels (rich vs. poor) and causes (merit vs. luck vs. uncertain) of wealth in our experiment design, and the resulting labels for different types of participants. Treatments were assigned at the group level such that a group in the merit treatment consisted of two deserving rich and two deserving poor players; a group in the luck treatment consisted of two lucky rich and two unlucky poor players; and a group in the uncertain treatment consisted of two uncertain rich and two uncertain poor players.			

**Table 2 tbl2:** Main Hypotheses

Hypothesis	Theme	Prediction
1	Fairness: insufficiency	Participants will on average judge it fair that richer and poorer players contribute less than 50% of their wealth;
2	Fairness: level of wealth (rich vs. poor)	Richer (poorer) participants will judge it fair that they contribute a lower proportion of their wealth than poorer (richer) participants;
3	Contributions: level of wealth (rich vs. poor)	Richer participants will contribute more than poorer participants in absolute terms, but less in proportional terms;
4	Fairness: cause of wealth (merit vs. luck vs. uncertain)	Individuals in the merit treatment will expect the poor to contribute a higher proportion of their wealth;
5	Contributions: cause of wealth (merit vs. luck vs. uncertain)	The deserving rich will contribute a lower proportion of their wealth than the uncertain and the lucky rich
6	Group outcomes	Luck-based groups would be more successful than merit-based and uncertain groups
*Note*. The table summarizes our main hypotheses, including the theme and our predictions for each.		

**Table 3 tbl3:** Hypotheses and Result*s*

Hypothesis	Theme	Prediction	Statistical tests	Supported
1	Fairness: insufficiency	Participants’ will on average judge it fair that richer and poorer players contribute less than 50% of their wealth	None	✔
2	Fairness: level of wealth (rich vs. poor)	Richer (poorer) participants will judge it fair that they contribute a lower proportion of their wealth than poorer (richer) participants	Chi-squared tests (H2a); Standard and Bayesian ANOVAs (H2b-c)	**∼**
3 (PR)	Contributions: level of wealth (rich vs. poor)	Richer participants will contribute more than poorer participants in absolute terms, but less in proportional terms	Standard and Bayesian ANOVAs + Multilevel Models	✔
4 (PR)	Fairness: cause of wealth (merit vs. luck vs. uncertain)	Individuals in the merit treatment will expect the poor to contribute a higher proportion of their wealth	Standard and Bayesian ANOVAs	✖
5 (PR)	Contributions: cause of wealth (merit vs. luck vs. uncertain)	The deserving rich will contribute a lower proportion of their wealth than the uncertain and the lucky rich	Standard and Bayesian ANOVAs + Multilevel Model	✖
6 (PR)	Group outcomes	Luck-based groups would be more successful than merit-based and uncertain groups	Chi-squared tests	✖
*Note*. The table summarizes the statistical tests we used for our main hypotheses (PR = preregistered) and whether they were supported. ANOVA = analysis of variance.				

## References

[ref1] Adams J. S. (1965). Inequity in social exchange. In Berkowitz L. (Ed.), Advances in experimental social psychology (pp. 267–299). Academic Press

[ref2] Alan S., & Ertac S. (2017). Belief in hard work and altruism: Evidence from a randomized field experiment (Working Paper No. 2017-053). Human Capital and Economic Opportunity Working Group.

[ref3] Alesina A., & Glaeser E. L. (2004). Fighting poverty in the US and Europe: A world of difference. Oxford University Press.

[ref4] Alesina A., Stantcheva S., & Teso E. (2018). Intergenerational mobility and preferences for redistribution. American Economic Review, 108(2), 521–554. 10.1257/aer.20162015

[ref5] Alesina A. F., Glaeser E. L., & Sacerdote B. (2001). Why doesn’t the US have a European-style welfare system? (Brookings Paper on Economic Activity No. 2). Brookings Institution.

[ref6] Babcock L., & Loewenstein G. (1997). Explaining bargaining impasse: The role of self-serving biases. Journal of Economic Perspectives, 11(1), 109–126. 10.1257/jep.11.1.109

[ref7] Baumeister R. F. (1982). A self-presentational view of social phenomena. Psychological Bulletin, 91(1), 3–26. 10.1037/0033-2909.91.1.3

[ref8] Bernard M., Reuben E., & Riedl A. (2014). Fairness and coordination: The role of fairness principles in coordination failure and success. Mimeo.

[ref9] Bowles S., & Gintis H. (2011). A cooperative species. Princeton University Press.

[ref10] Brick K., & Visser M. (2015). What is fair? An experimental guide to climate negotiations. European Economic Review, 74, 79–95. 10.1016/j.euroecorev.2014.11.010

[ref11] Camerer C. F. (2003). Behavioral game theory: Experiments in strategic interaction. Princeton University Press.

[ref12] Cappelen A. W., Mollerstrom J., Reme B. A., & Tungodden B. (2022). A meritocratic origin of egalitarian behaviour. The Economic Journal, 132(646), 2101–2117. 10.1093/ej/ueac008

[ref13] Carlsson F., Kataria M., Krupnick A., Lampi E., Löfgren Å, Qin P., & Sterner T. (2013). A fair share: Burden-sharing preferences in the United States and China. Resource and Energy Economics, 35(1), 1–17. 10.1016/j.reseneeco.2012.11.001

[ref14] Carpenter G., Kleinjans R., Villasante S., & O’Leary B. C. (2016). Landing the blame: The influence of EU member states on quota setting. Marine Policy, 64(26), 9–15. 10.1016/j.marpol.2015.11.001

[ref15] Chan K. S., Mestelman S., Moir R., & Muller R. A. (1996). The voluntary provision of public goods under varying income distributions. Canadian Journal of Economics, 29(1), 54–69. 10.2307/136151

[ref16] Chen D. L., Schonger M., & Wickens C. (2016). oTree—An open-source platform for laboratory, online, and field experiments. Journal of Behavioral and Experimental Finance, 9, 88–97. 10.1016/j.jbef.2015.12.001

[ref17] Croson R., Fatas E., & Neugebauer T. (2005). Reciprocity, matching and conditional cooperation in two public goods games. Economics Letters, 87(1), 95–101. 10.1016/j.econlet.2004.10.007

[ref18] Davide M., Parrado R., & Campagnolo L. (2017). Fairness in NDCs: Comparing mitigation efforts from an equity perspective. United Nations Framework Convention on Climate Change. https://unfccc.int/sites/default/files/resource/312_Fairness%20in%20NDCs.%20Comparing%20mitigation%20efforts%20from%20an%20equity%20perspective.pdf

[ref19] De Cremer D. (2007). When the rich contribute more in public good dilemmas: The role of provision point level. European Journal of Social Psychology, 37(3), 536–546. 10.1002/ejsp.368

[ref20] Diekmann K. A., Samuels S. M., Ross L., & Bazerman M. H. (1997). Self-interest and fairness in problems of resource allocation: Allocators versus recipients. Journal of Personality and Social Psychology, 72(5), 1061–1074. 10.1037/0022-3514.72.5.1061 9150585

[ref21] Elster J. (2006). Fairness and norms. Social Research, 73(2), 365–376. 10.1353/sor.2006.0033

[ref22] Fehr E., & Schmidt K. M. (1999). A theory of fairness, competition, and cooperation. The Quarterly Journal of Economics, 114(3), 817–868. 10.1162/003355399556151

[ref23] Fischbacher U., & Gächter S. (2010). Social preferences, beliefs, and the dynamics of free riding in public goods experiments. American Economic Review, 100(1), 541–556. 10.1257/aer.100.1.541

[ref24] Fischbacher U., Gächter S., & Fehr E. (2001). Are people conditionally cooperative? Evidence from a public goods experiment. Economics Letters, 71(3), 397–404. 10.1016/S0165-1765(01)00394-9

[ref25] Flusfeder D. (2022). Luck: A personal account of fortune, chance and risk in 13 investigations. 4th Estate.

[ref26] Fong C. (2001). Social preferences, self-interest, and the demand for redistribution. Journal of Public Economics, 82(2), 225–246. 10.1016/S0047-2727(00)00141-9

[ref27] Frank R. H. (2016). Success and luck. Princeton University Press.

[ref28] Gächter S. (2006). Conditional cooperation: Behavioral regularities from the lab and the field and their policy implications (CeDEx Discussion Paper Series No. 2006-03). Centre for Decision Research and Experimental Economics.

[ref29] Gonzalez A. M., Macchia L., & Whillans A. V. (2022). The developmental origins and behavioral consequences of attributions for inequality. Journal of Experimental Social Psychology, 101(6323), Article 104329. 10.1016/j.jesp.2022.104329

[ref30] Hardin G. (1968). The tragedy of the commons. Science, 162(3859), 1243–1248. 10.1126/science.162.3859.1243 5699198

[ref31] Hargreaves Heap S. P. H., Ramalingam A., & Stoddard B. V. (2016). Endowment inequality in public goods games: A re-examination. Economics Letters, 146, 4–7. 10.1016/j.econlet.2016.07.015

[ref32] Henrich N., & Henrich J. P. (2007). Why humans cooperate: A cultural and evolutionary explanation. Oxford University Press.

[ref33] Hine D. W., & Gifford R. (1996). Attributions about self and others in commons dilemmas. European Journal of Social Psychology, 26(3), 429–445. 10.1002/(SICI)1099-0992(199605)26:3<429::AID-EJSP767>3.0.CO;2-P

[ref34] Hume D. (1740). A treatise of human nature. Clarendon Press.

[ref35] JASP Team. (2020). JASP (Version 0.17.2) [Computer software]. https://jasp-stats.org/

[ref36] Joireman J. A., Kuhlman D. M., & Okuda H. (1994). Fairness judgements in an asymmetric public goods dilemma. In Schulz U., Albers W., & Mueller U. (Eds.), Social dilemmas and cooperation (pp. 99–116). Springer-Verlag.

[ref37] Kelley H. H., & Stahelski A. J. (1970). Social interaction basis of cooperators’ and competitors’ beliefs about others. Journal of Personality and Social Psychology, 16(1), 66–91. 10.1037/h0029849

[ref38] Keser C., & Van Winden F. (2000). Conditional cooperation and voluntary contributions to public goods. Scandinavian Journal of Economics, 102(1), 23–39. 10.1111/1467-9442.00182

[ref39] Konow J. (2000). Fair shares: Accountability and cognitive dissonance in allocation decisions. American Economic Review, 90(4), 1072–1092. 10.1257/aer.90.4.1072

[ref40] Koo H. J., Piff P. K., & Shariff A. F. (2022). If I could do it, so can they: Among the rich, those with humbler origins are less sensitive to the difficulties of the poor. Social Psychological and Personality Science, 14(3), 333–341. 10.1177/19485506221098921 36844784 PMC9947719

[ref41] Kopelman S., Weber J. M., & Messick D. M. (2002). Factors influencing cooperation in commons dilemmas: A review of experimental psychological research. In Ostrom E., Dietz T., Dolšak N., Stern P. C., Stonich S., Weber E. U., & Committee on the Human Dimensions of Global Change, Division of Behavioral and Social Sciences and Education (Eds.), The drama of the commons (pp. 113–156). National Academy Press.

[ref42] Laming D. (1984). The relativity of ‘absolute’ judgements. British Journal of Mathematical and Statistical Psychology, 37(2), 152–183. 10.1111/j.2044-8317.1984.tb00798.x

[ref43] Lange A., Löschel A., Vogt C., & Ziegler A. (2010). On the self-interested use of equity in international climate negotiations. European Economic Review, 54(3), 359–375. 10.1016/j.euroecorev.2009.08.006

[ref44] Ledyard J. O. (1995). Public goods: A survey of experimental research. In Kagel J. H. & Roth A. E. (Eds.), Handbook of experimental economics (Vol. 2, pp. 111–181). Princeton University Press.

[ref45] Litman L., Moss A., Rosenzweig C., & Robinson J. (2021). Reply to MTurk, prolific or panels? Choosing the right audience for online research. Choosing the right audience for online research (January 28, 2021). https://ssrn.com/abstract=3775075 or 10.2139/ssrn.3775075

[ref46] Markovits D. (2019). The meritocracy trap. Penguin UK.

[ref47] Martinangeli A. F., & Martinsson P. (2020). We, the rich: Inequality, identity and cooperation. Journal of Economic Behavior & Organization, 178(3), 249–266. 10.1016/j.jebo.2020.07.013

[ref48] Marwell G., & Ames R. E. (1979). Experiments on the provision of public goods. I. Resources, interest, group size, and the free-rider problem. American Journal of Sociology, 84(6), 1335–1360. 10.1086/226937

[ref49] Milinski M., Sommerfeld R. D., Krambeck H. J., Reed F. A., & Marotzke J. (2008). The collective-risk social dilemma and the prevention of simulated dangerous climate change. Proceedings of the National Academy of Sciences, 105(7), 2291–2294. 10.1073/pnas.0709546105 PMC2268129 18287081

[ref50] Miller D. T., & Ross M. (1975). Self-serving biases in the attribution of causality: Fact or fiction? Psychological Bulletin, 82(2), 213–225. 10.1037/h0076486

[ref51] Niederle M., & Vesterlund L. (2007). Do women shy away from competition? Do men compete too much? The Quarterly Journal of Economics, 122(3), 1067–1101. 10.1162/qjec.122.3.1067

[ref52] Nowak M., & Highfield R. (2011). Supercooperators: Altruism, evolution, and why we need each other to succeed. Simon & Schuster.

[ref53] Olson M. (1965). The logic of collective action: Public goods and the theory of groups. Harvard University Press.

[ref54] Ostrom E. (2008). Tragedy of the commons. In Durlauf S. N. & Blume L. E. (Eds.), The new Palgrave dictionary of economics (2nd ed.). Palgrave Macmillan. 10.1057/978-1-349-95121-5_2047-1

[ref55] Ostrom E. (2010). Analyzing collective action. Agricultural Economics, 41, 155–166. 10.1111/j.1574-0862.2010.00497.x

[ref56] Oswald A. J., Proto E., & Sgroi D. (2015). Happiness and productivity. Journal of Labor Economics, 33(4), 789–822. 10.1086/681096

[ref57] Palan S., & Schitter C. (2018). Prolific. ac—A subject pool for online experiments. Journal of Behavioral and Experimental Finance, 17, 22–27. 10.1016/j.jbef.2017.12.004

[ref58] Paolacci G., Chandler J., & Ipeirotis P. G. (2010). Running experiments on Amazon mechanical Turk. Judgement and Decision Making, 5(5), 411–419. 10.1017/S1930297500002205

[ref59] Piketty T. (1995). Social mobility and redistributive politics. The Quarterly Journal of Economics, 110(3), 551–584. 10.2307/2946692

[ref60] Piketty T. (2020). Capital and ideology. Harvard University Press.

[ref61] Rabin M. (1993). Incorporating fairness into game theory and economics. American Economic Review, 83, 1281–1302. 10.2307/j.ctvcm4j8j.15

[ref62] Rapoport A. (1988). Provision of step-level public goods: Effects of inequality in resources. Journal of Personality and Social Psychology, 54(3), 432–440. 10.1037/0022-3514.54.3.432

[ref63] Reindl I. (2022). Wealth and vulnerability to climate change: An experimental study on burden sharing among heterogeneous agents. Environmental and Resource Economics, 82(4), 791–823. 10.1007/s10640-022-00672-3

[ref64] Samuelson C. D., & Allison S. T. (1994). Cognitive factors affecting the use of social decision heuristics in resource-sharing tasks. Organizational Behavior and Human Decision Processes, 58(1), 1–27. 10.1006/obhd.1994.1027

[ref65] Sandel M. J. (2020). The tyranny of merit: What’s become of the common good? Penguin UK.

[ref66] Sandler T. (1992). Collective action: Theory and applications. University of Michigan Press.

[ref67] Son Hing L. S., Bobocel D. R., Zanna M. P., Garcia D. M., Gee S. S., & & Orazietti K. (2011). The merit of meritocracy. Journal of Personality and Social Psychology, 101(3), 433–450. 10.1037/a0024618 21787093

[ref68] Starmans C., Sheskin M., & Bloom P. (2017). Why people prefer unequal societies. Nature Human Behaviour, 1(4), 1–7. 10.1038/s41562-017-0082

[ref69] Stewart N., Brown G. D., & Chater N. (2005). Absolute identification by relative judgment. Psychological Review, 112(4), 881–911. 10.1037/0033-295X.112.4.881 16262472

[ref70] Stewart N., Chater N., & Brown G. D. (2006). Decision by sampling. Cognitive Psychology, 53(1), 1–26. 10.1016/j.cogpsych.2005.10.003 16438947

[ref71] Stroebe W., & Frey B. S. (1982). Self-interest and collective action: The economics and psychology of public goods. British Journal of Social Psychology, 21(2), 121–137. 10.1111/j.2044-8309.1982.tb00521.x

[ref72] Sugden R. (1984). Reciprocity: The supply of public goods through voluntary contributions. The Economic Journal, 94(376), 772–787. 10.2307/2232294

[ref73] Tavoni A., Dannenberg A., Kallis G., & Löschel A. (2011). Inequality, communication, and the avoidance of disastrous climate change in a public goods game. Proceedings of the National Academy of Sciences, 108(29), 11825–11829. 10.1073/pnas.1102493108 PMC3141931 21730154

[ref74] The Sunday Times. (2021). Marcus Rashford tops the Sunday Times Giving List 2021. https://www.thetimes.co.uk/article/marcus-rashford-sunday-times-giving-list-2021-l9td90n0l

[ref75] United Nations. (2015). Paris agreement. https://unfccc.int/process-and-meetings/the-paris-agreement/the-paris-agreement

[ref76] United Nations Framework Convention on Climate Change. (2021). https://unfccc.int/news/cop26-update-to-the-ndc-synthesis-report

[ref77] Van Dijk E., & Wilke H. (1993). Differential interests, equity, and public good provision. Journal of Experimental Social Psychology, 29(1), 1–16. 10.1006/jesp.1993.1001

[ref78] Van Dijk E., & Wilke H. (1994). Asymmetry of wealth and public good provision. Social Psychology Quarterly, 57, 352–359.

[ref79] Van Lange P. A., Joireman J., Parks C. D., & Van Dijk E. (2013). The psychology of social dilemmas: A review. Organizational Behavior and Human Decision Processes, 120(2), 125–141. 10.1016/j.obhdp.2012.11.003

[ref80] Van Segbroeck S., Pacheco J. M., Lenaerts T., & Santos F. C. (2012). Emergence of fairness in repeated group interactions. Physical Review Letters, 108(15), Article 158104. 22587290 10.1103/PhysRevLett.108.158104

[ref81] Vasconcelos V. V., Santos F. C., Pacheco J. M., & Levin S. A. (2014). Climate policies under wealth inequality. Proceedings of the National Academy of Sciences, 111(6), 2212–2216. 10.1073/pnas.1323479111 PMC3926044 24469806

[ref82] Vicens J., Bueno-Guerra N., Gutiérrez-Roig M., Gracia-Lázaro C., Gómez-Gardeñes J., Perelló J., Sánchez A., Moreno Y., & Duch J. (2018). Resource heterogeneity leads to unjust effort distribution in climate change mitigation. PLoS One, 13(10), Article e0204369. 10.1371/journal.pone.0204369 30379845 PMC6209147

[ref83] Wade-Benzoni K. A., Tenbrunsel A. E., & Bazerman M. H. (1996). Egocentric interpretations of fairness in asymmetric, environmental social dilemmas: Explaining harvesting behavior and the role of communication. Organizational Behavior and Human Decision Processes, 67(2), 111–126. 10.1006/obhd.1996.0068

[ref84] Walster E., Berscheid E., & Walster G. W. (1976). New directions in equity research. In Berkowitz L. & Walster E. (Eds.), Advances in experimental social psychology (Vol. 9, pp. 1–42). Academic Press. 10.1016/S0065-2601(08)60057-X

