# Correction to “When Fairness Is Not Enough: The Disproportionate Contributions of the Poor in a Collective Action Problem” by Malthouse et al. (2023)

**DOI:** 10.1037/xge0001631

**Published:** 2024-07

In the article “When Fairness Is Not Enough: The Disproportionate Contributions of the Poor in a Collective Action Problem” by Eugene Malthouse, Charlie Pilgrim, Daniel Sgroi, and Thomas T. Hills (*Journal of Experimental Psychology: General*, 2023, Vol. 152, No. 11, pp. 3229–3242, https://doi.org/10.1037/xge0001455), the third and final research question in The Collective-Risk Social Dilemma section now appears as follows: 3. If what people perceive as fair is insufficient to solve the problem, under what conditions do groups still manage to succeed? The online version of this article has been corrected.

