# Supplementary material for: When Fairness Is Not Enough: The Disproportionate Contributions of the Poor in a Collective Action Problem
Source: J Exp Psychol Gen. 2023 Jul 20;152(11):3229–42. doi: 10.1037/xge0001455 (PMC10585937; doi:10.1037/xge0001455)
Supplement: Supplementary file 1 [file xge0001455.pdf]

Supplementary Materials

Supplementary Figures

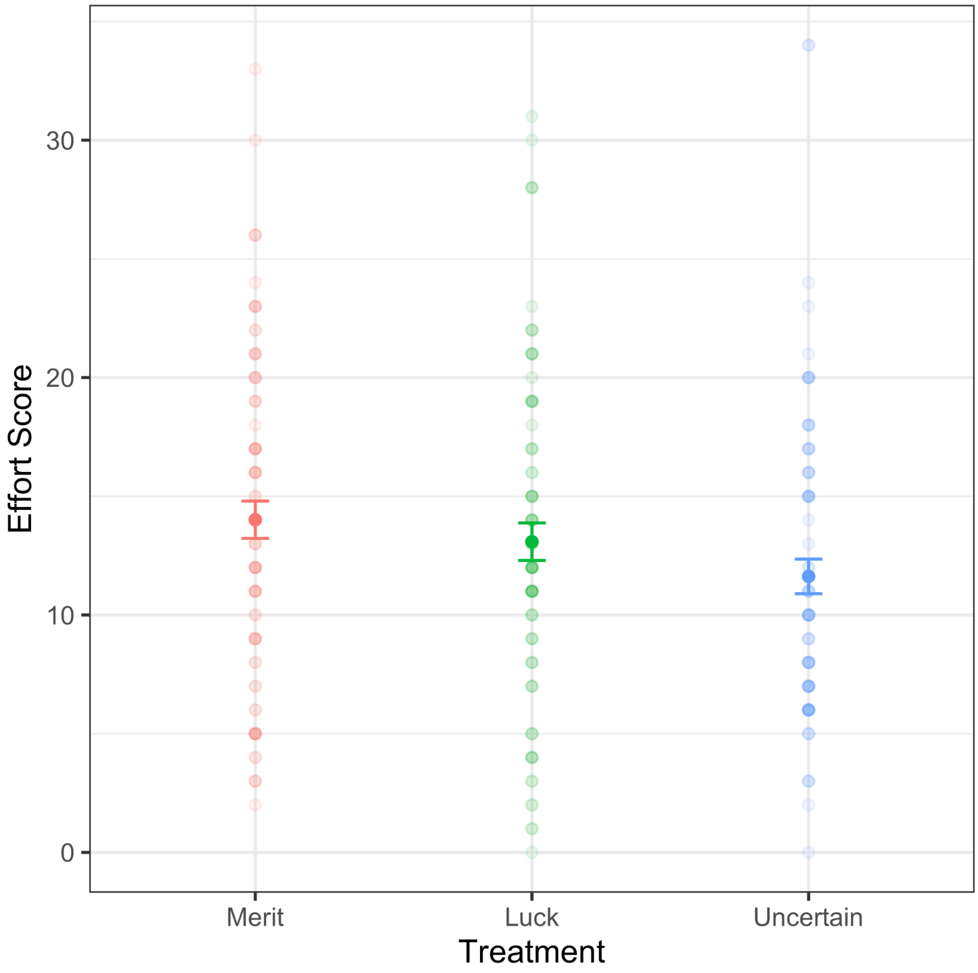

Figure S1. *Effort Scores Between Treatment Conditions*

Participants' scores in our five-minute effort task, split by treatment. Coloured points represent the mean, with coloured bars representing the standard error. Grey points in the background represent raw data. Differences between treatments suggest that participants understood the different implications of their performance in each condition, but we did not detect a significant treatment effect $F(2, 237) = 2.5$, $p = .088$.

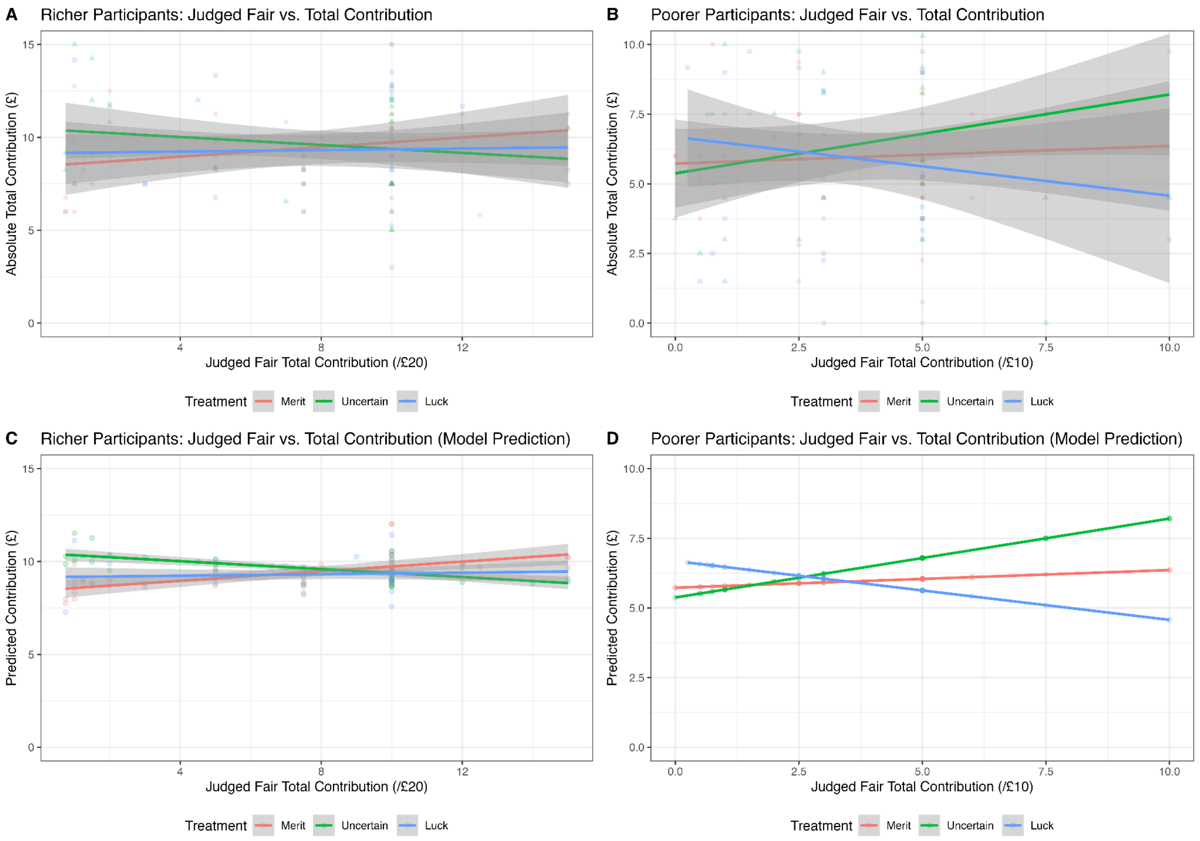

Figure S2. *Contributions as a Function of Fairness Judgements*

Plots illustrate the relationship between: x) richer and poorer participants' judgements about what was fair for someone of their wealth level to contribute; and y) their total contribution (their actual total contribution in plots A and B vs. their contribution predicted by our model in plots C and D). Plots A and B show the relationship between participants' judgements and what they actually contributed within the game in absolute terms, with points in the background representing individuals, separate linear regression lines for each treatment, and shaded areas representing the standard error. Plots C and D show predicted contributions from multilevel models that included total contribution as the dependent variable, fixed fairness judgement and treatment effects, and random group effects to take account of the nested structure of the data. These models did not detect any effect of fairness judgements for either richer participants ($t(107) = 0.7$, $p = .513$) or poorer participants ($t(112) = 0.4$, $p = .735$). Points in the background represent predictions for individuals and coloured lines are generalised linear regression lines for each treatment.

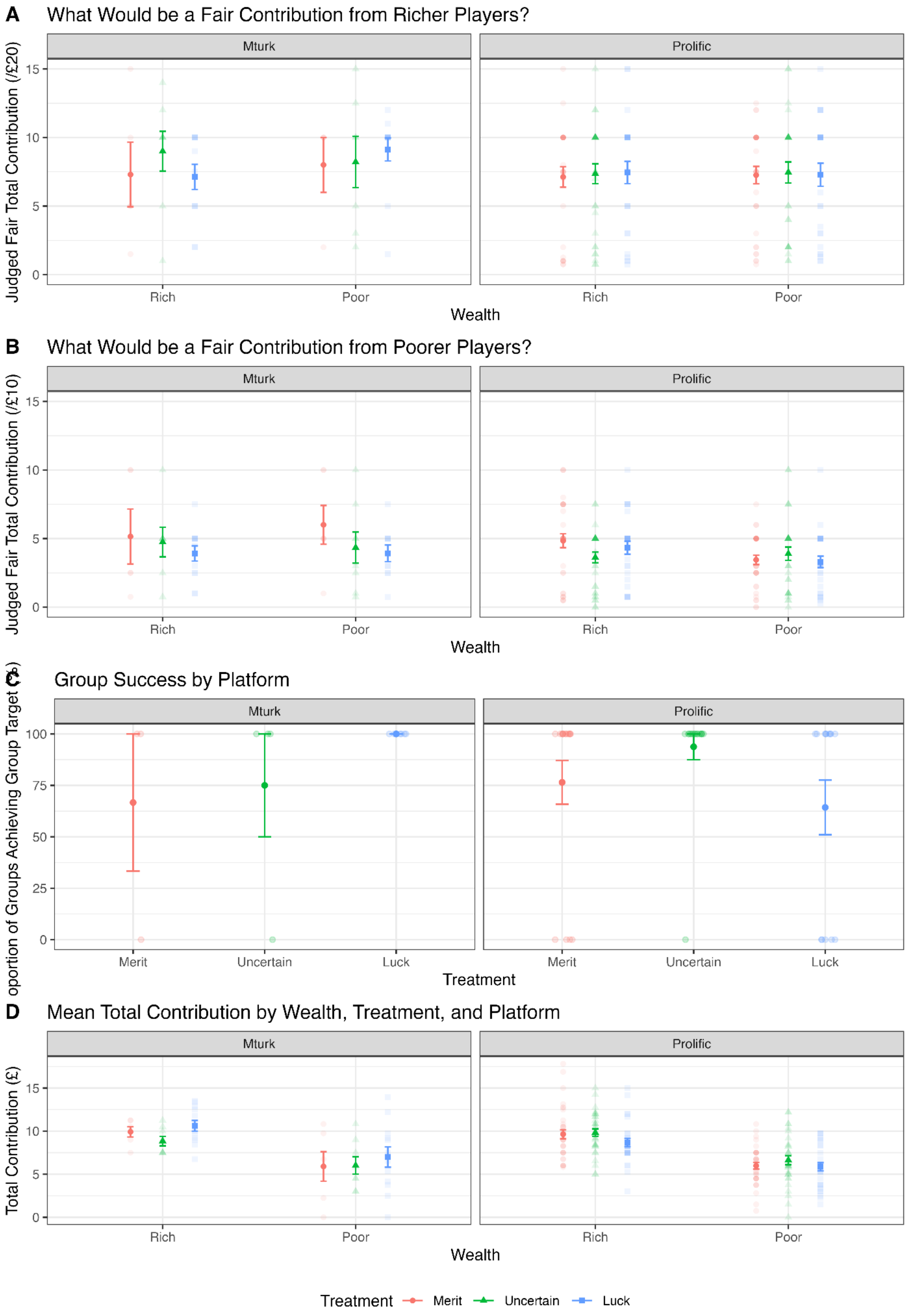
A
What Would be a Fair Contribution from Richer Players?
Mturk
Prolific
Judged Fair Total Contribution (/£20)
0
5
10
15
Rich
Poor
Wealth
B
What Would be a Fair Contribution from Poorer Players?
Mturk
Prolific
Judged Fair Total Contribution (/£10)
0
5
10
15
Rich
Poor
Wealth
C
Group Success by Platform
Mturk
Prolific
oportion of Groups Achieving Group Target (%)
0
25
50
75
100
Merit
Uncertain
Luck
Treatment
D
Mean Total Contribution by Wealth, Treatment, and Platform
Mturk
Prolific
Total Contribution (£)
0
5
10
15
Rich
Poor
Wealth
Treatment
Merit
Uncertain
Luck

Figure S3. *Main Outcome Variables by Data Collection Platform (MTurk and Prolific)*

Plots A and B show responses to the two questions put to participants in the pre-game questionnaire: ‘In your opinion, what would be a fair total contribution in £ to the group account during the game?’ for players starting with £20 (plot A) and players starting with £10 (plot B). ANOVAs did not detect a platform effect for judgements about richer players ($F(1, 216) = 1.31$, $p = .254$) or poorer players ($F(1, 224) = 3.32$, $p = .070$). Points in the background represent individuals, with summary points showing the mean response and error bars representing the standard error. Plot C shows the proportion of groups who achieved the target in each treatment, split by platform. Points in the background represent groups. Summary points show the mean success rate where 100 represents group success and 0 represents group failure, with error bars representing the standard error. Chi-squared tests indicated that the proportion of groups that were successful in each treatment did not differ significantly between platforms (merit: $\chi^2(1) = 0$, $p = 1$; luck: $\chi^2(1) = 1.27$, $p = .260$; uncertain: $\chi^2(1) = 0.03$, $p = .852$). Plot D shows absolute contributions to the group account by wealth and treatment levels and platform, with points in the background representing individuals and summary points showing the mean response (and error bars represent the standard error). An ANOVA did not detect a significant platform effect ($F(1, 228) = 0.40$, $p = .526$).

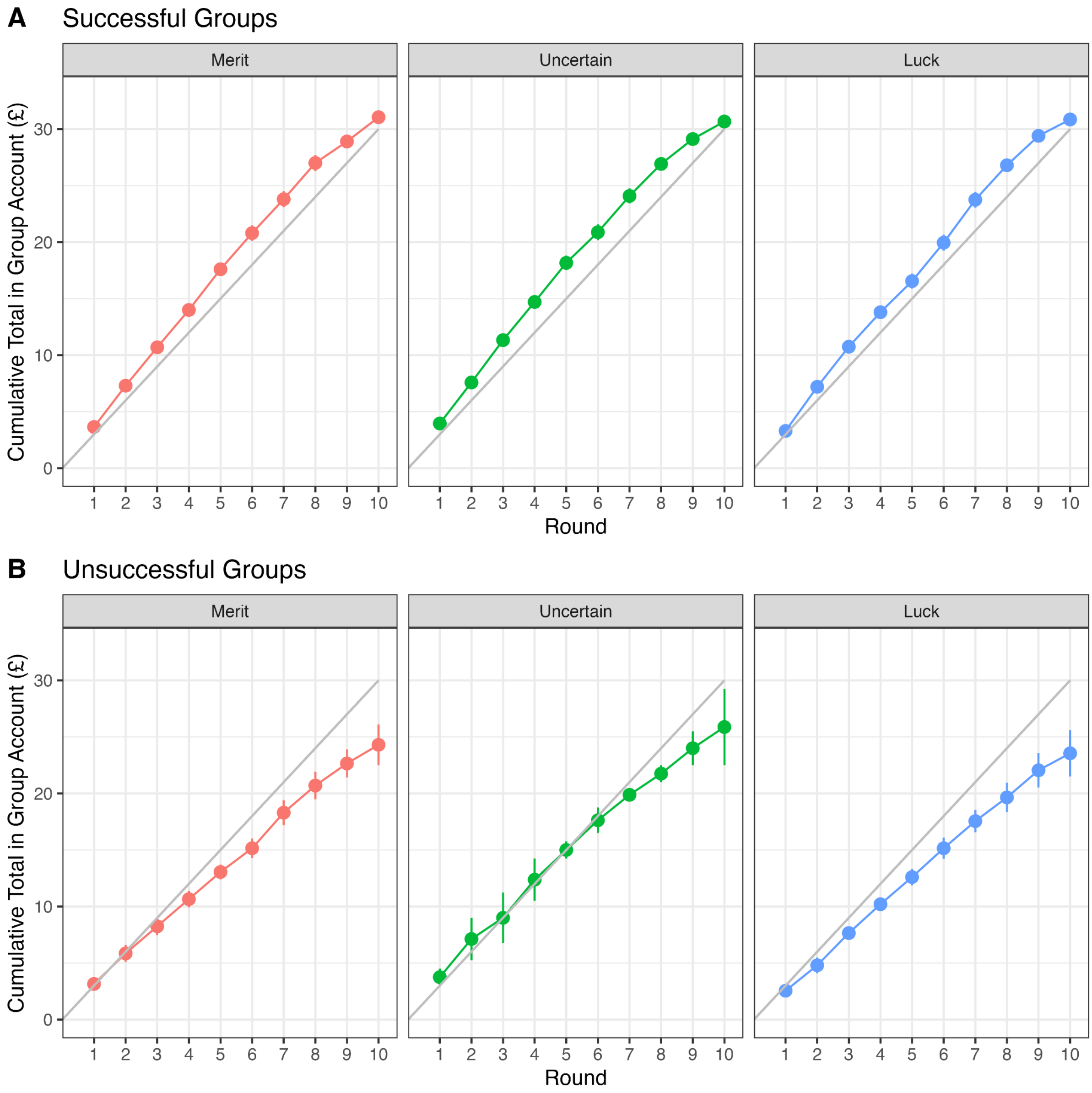

Figure S4. *Cumulative Group Contributions Between Treatments*

Plots show the cumulative total in group accounts in successive rounds. Groups are divided by outcome and treatment, with A plots showing successful groups and B plots showing unsuccessful groups by treatment. Coloured points show the mean cumulative total in the group account in each round, with bars representing the standard error. Grey lines show a linear trajectory towards achieving the group target of £30 within the 10 rounds.

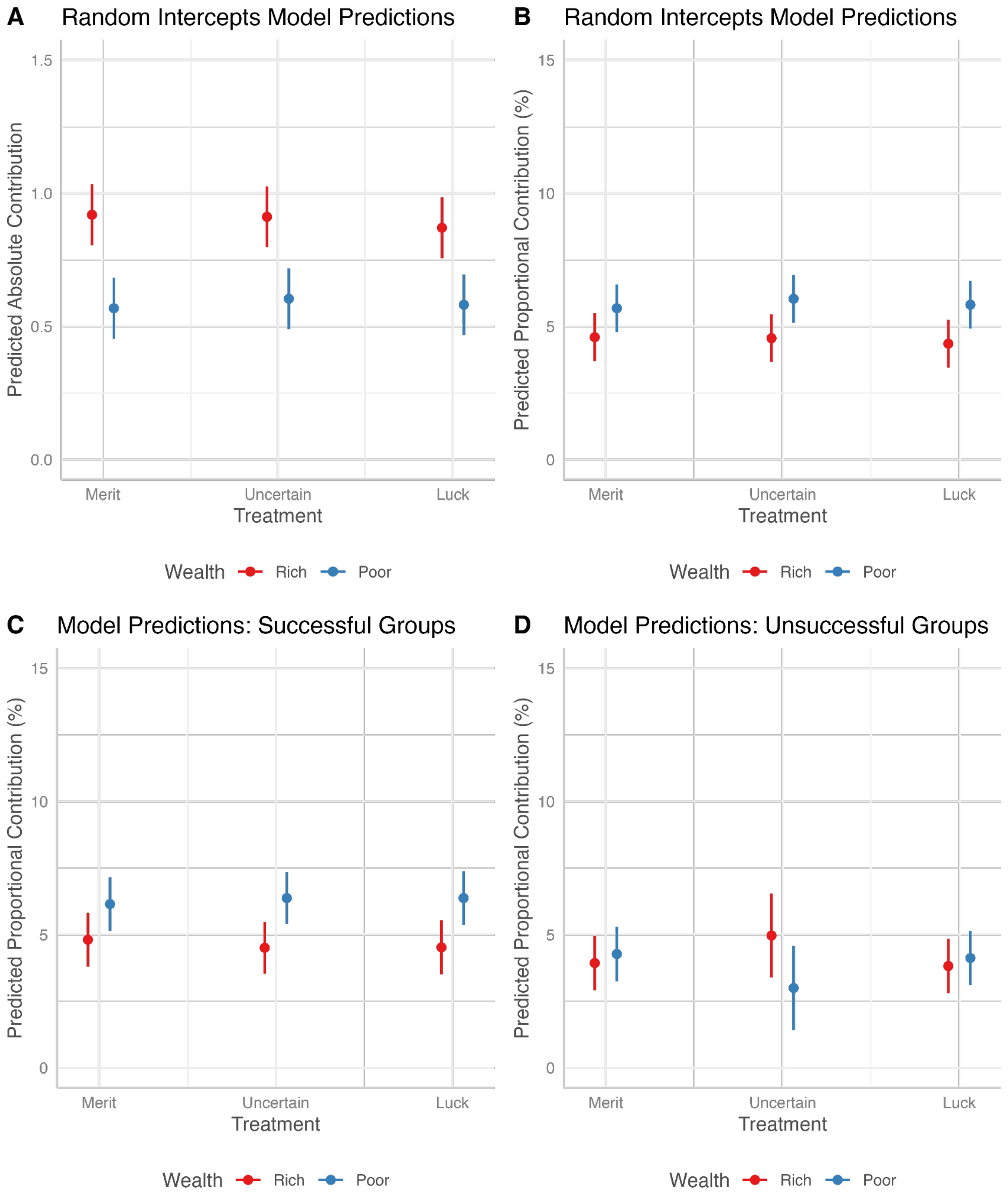

Figure S5. *Multilevel Model Predictions: Contributions by Treatment, Wealth, and Group Success*

Plots show predicted contributions from multilevel models with absolute and relative contributions specified as the dependent variable and treatment and wealth as predictor variables, with random intercepts at the round, individual, and group levels (see full model outputs in Table 1 below). Plot A shows predicted absolute contributions. Plot B shows predicted contributions in relative terms. Plot C shows predicted relative contributions

in successful groups, showing higher predictions for poorer participants across all treatments. Plot D shows predicted relative contributions in unsuccessful groups.

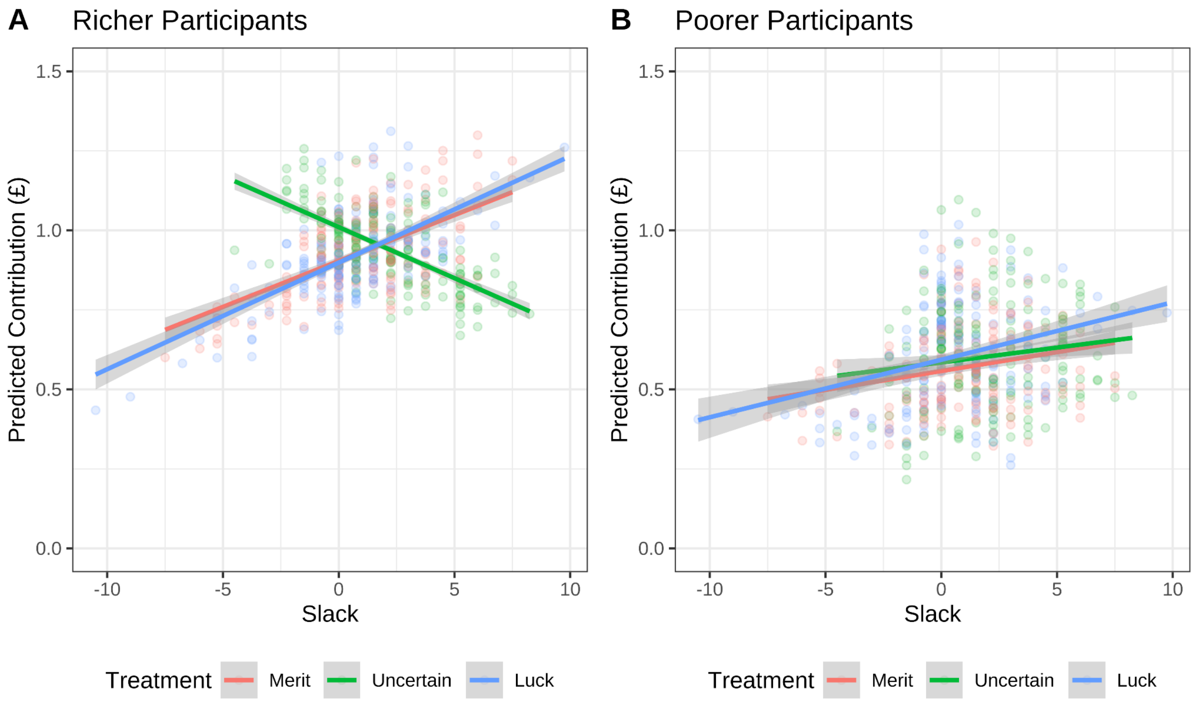

Figure S6. *Multilevel Model Predictions: Contributions as a Function of Slack*

Plots show predicted individual absolute contributions from richer participants (A) and poorer participants (B) as a function of slack (defined as the difference between £3 per round cumulative required contribution and the current group total) , with separate slopes for different treatment levels. Multilevel models included fixed treatment and slack effects and random intercepts at the round and group level. The plots illustrate the generally positive relationship between the level of slack and predicted contributions – with the exception of richer participants in the uncertain treatment, who *increase* their contributions when the group falls behind the required rate.

Supplementary Tables

Table S1. *Relative Contributions: Multilevel Model Parameter Estimates*

The table shows estimates from a multilevel model with relative contributions as the dependent variable, treatment and wealth as fixed effects, and random intercepts at the round, individual, and group levels. The fixed part of the model indicates a significant wealth effect. The random part of the model shows that a greater proportion of variance is accounted for at the round level (6.7%) than at the group level (0.7%).

	**Estimate**	**Standard Err.**	**Degrees of Freedom**	***t*-value**	***p*-value**
**Fixed Effects**					
Intercept (Merit Treatment)	4.59	0.46	27	10.05	< .001
Uncertain Treatment	-0.04	0.44	234	-0.09	.932
Luck Treatment	-0.24	0.44	234	-0.56	.578
Wealth Poor	1.09	0.44	234	2.49	.014*
Luck Treatment: Wealth Poor	0.39	0.62	234	0.64	.525
Uncertain Treatment: Wealth Poor	0.38	0.62	234	0.61	.545
**Random Effects**	**Variance**	**Standard Dev.**			
Intercept (UniqueID)	2.51	1.58			
Intercept (GroupID)	0.00	0.00			
Intercept (Round)	1.13	1.07			
Residual	13.15	3.63			

Table S2. *Contributions as a Function of Slack: Multilevel Model Parameter Estimates*

The table shows estimates from multilevel models on data from richer and poorer participants with absolute contributions as dependent variables, treatment and slack as fixed effects, and random intercepts at the round individual, and group levels.

	**Estimate**	**Standard Err.**	**Degrees of Freedom**	***t*-value**	***p*-value**
**Richer Participants**					
**Fixed Effects**					
Intercept (Merit Treatment)	0.92	0.05	40	19.71	< .001
Uncertain Treatment	0.09	0.07	54	1.44	.155
Luck Treatment	-0.02	0.06	45	-0.30	.767
Slack	0.01	0.01	158	0.47	.638
Uncertain Treatment: Slack	-0.46	0.02	168	-2.47	.015*
Luck Treatment: Slack	0.01	0.02	161	0.59	.559
**Random Effects**	**Variance**	**Standard Dev.**			
Intercept (UniqueID)	0.02	0.16			
Intercept (GroupID)	0.01	0.09			
Intercept (Round)	0.00	0.06			
Residual	0.21	0.46			
**Poorer Participants**					
**Fixed Effects**					
Intercept (Merit Treatment)	0.59	0.06	40	9.57	< .001
Uncertain Treatment	0.07	0.08	44	0.85	.403
Luck Treatment	0.02	0.08	37	0.25	.802
Slack	-0.03	0.01	23	-2.00	.046*
Uncertain Treatment: Slack	-0.00	0.02	25	-0.04	.966
Luck Treatment: Slack	0.01	0.02	24	0.38	.704
**Random Effects**	**Variance**	**Standard Dev.**			

Intercept (UniqueID)	0.06	0.25
Intercept (GroupID)	0.01	0.11
Intercept (Round)	0.01	0.09
Residual	0.20	0.44

Table S3. *Prolific Academic Participant Backgrounds*

The table summarises the demographic data (ethnicity and nationality) that were retrospectively available for 180 participants sampled from Prolific Academic. It therefore does not reflect our full sample of 240 participants, and should only be treated as an approximate indication of the make-up of our sample population.

	**Count**	**%**		**Count**	**%**
**Ethnicity**			**Nationality**		
Asian	7	4%	Algeria	1	0.6%
Black	17	9%	Australia	1	0.6%
Mixed	16	9%	Austria	1	0.6%
Other	5	3%	Canada	3	1.7%
White	133	74%	Czech Republic	1	0.6%
Missing	2	1%	Egypt	1	0.6%
			Estonia	1	0.6%
			France	2	1.1%
			Germany	1	0.6%
			Greece	2	1.1%
			Hungary	4	2.2%
			Iran	1	0.6%
			Ireland	1	0.6%
			Italy	12	6.7%
			Korea	1	0.6%
			Lebanon	1	0.6%
			Mexico	10	5.6%
			Netherlands	4	2.2%
			Poland	25	13.9%
			Portugal	29	16.1%
			Slovenia	1	0.6%
			South Africa	20	11.1%
			Spain	5	2.8%

			Turkey	1	0.6%
			United Kingdom	41	22.8%
			United States	10	5.6%

Experiment Materials

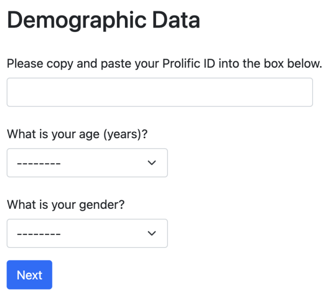
Demographic Data

Please copy and paste your Prolific ID into the box below.

What is your age (years)?

What is your gender?

Next

Figure S7. *Demographic Questions*

Drop-down options for age were: 18-24, 25-34, 35-44, 45-54, 55-64, 65+, I'd prefer not to answer. Drop-down options for gender were: Female, Male, Non-binary, Other, I'd prefer not to answer

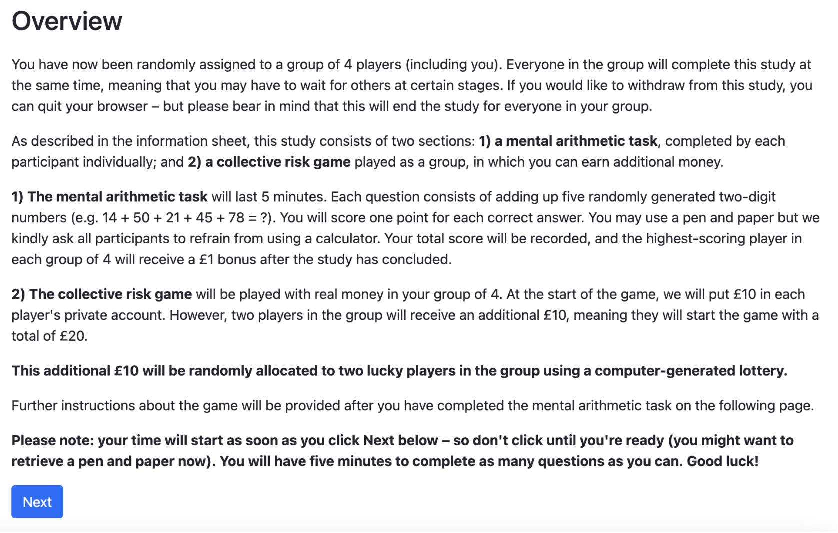
Overview

You have now been randomly assigned to a group of 4 players (including you). Everyone in the group will complete this study at the same time, meaning that you may have to wait for others at certain stages. If you would like to withdraw from this study, you can quit your browser – but please bear in mind that this will end the study for everyone in your group.

As described in the information sheet, this study consists of two sections: **1) a mental arithmetic task**, completed by each participant individually; and **2) a collective risk game** played as a group, in which you can earn additional money.

1) The mental arithmetic task will last 5 minutes. Each question consists of adding up five randomly generated two-digit numbers (e.g. 14 + 50 + 21 + 45 + 78 = ?). You will score one point for each correct answer. You may use a pen and paper but we kindly ask all participants to refrain from using a calculator. Your total score will be recorded, and the highest-scoring player in each group of 4 will receive a £1 bonus after the study has concluded.

2) The collective risk game will be played with real money in your group of 4. At the start of the game, we will put £10 in each player's private account. However, two players in the group will receive an additional £10, meaning they will start the game with a total of £20.

This additional £10 will be randomly allocated to two lucky players in the group using a computer-generated lottery.

Further instructions about the game will be provided after you have completed the mental arithmetic task on the following page.

Please note: your time will start as soon as you click Next below – so don't click until you're ready (you might want to retrieve a pen and paper now). You will have five minutes to complete as many questions as you can. Good luck!

Next

Figure S8. *Experiment Overview*

Screenshot shows the overview presented to participants in the luck treatment, with the explanation that "This additional £10 will be randomly allocated to two lucky players in the group using a computer-generated lottery." In the merit treatment, this equivalent sentence read: "This additional £10 will be given to the two players who score highest in each group in the mental arithmetic task". In the uncertain treatment, this sentence read: "Whether or

not you are one of the players who receives this additional £10 will be determined either by your performance in the mental arithmetic task or by chance – this will be randomly decided by the computer."

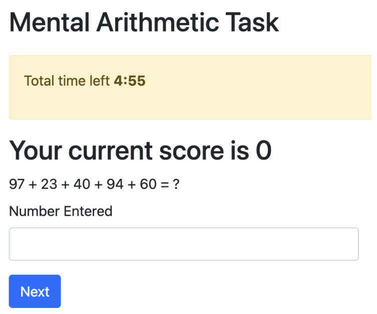

Figure S9. *Mental Arithmetic Task*

Experiment screenshot shows the mental arithmetic task, which lasted five minutes. Participants scored one mark for each correct answer. All two-digit numbers in the questions were randomly generated.

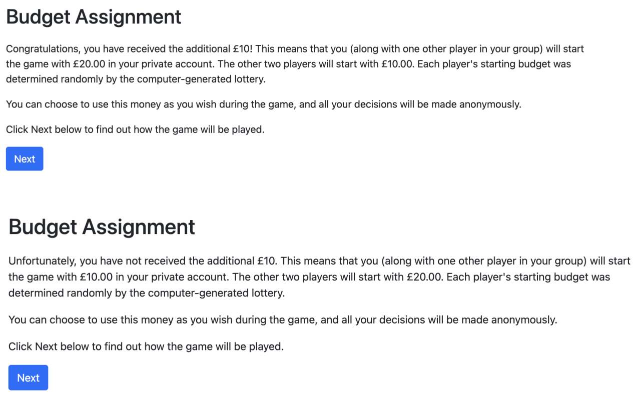

Figure S10. *Budget Assignment*

Screenshots shows budget assignments for richer participants (right) who receive the additional £10 and start the game with £20; and for poorer participants (right) who start the game with just £10.

Pre-Game Quiz

Please answer the following questions. The correct answers to the first three questions about the game instructions will be revealed on the following page.

Question 1

On average, how much in total (in £) over 10 rounds would each player have to transfer to the group account for the group to achieve its target of £30?

○ 0 ○ 3 ○ 7.5 ○ 10

Question 2

Imagine that the group succeeds in reaching the target of £30 after the 10 rounds, and you have £5 remaining in your private account. How much in £ will you take home from the game?

○ 0 ○ 2.5 ○ 5 ○ 10

Question 3

Assume that the group target of £30 is not reached, and you have £5 remaining in your private account. What is the % chance that you will lose this remaining amount in your private account?

○ 0 ○ 10 ○ 50 ○ 100

Question 4

In your opinion, what would be a fair total contribution in £ from players starting the game with £20 and those starting with £10? Please write your answer as a number (without the £ sign).

£20?

£10?

Next

Correct answers

Well done, you got the understanding questions correct. To double-check, here are the correct answers:

1) For the group to achieve its target of £30, on average each player should invest a total of **£7.50** to the group account (an average £0.75 on each round).

2) If the group account reaches the target of £30, and you have £5 remaining in your private account, you will take home **£5**.

3) If the group account does not reach the target of £30, and you have £5 remaining in your private account, you will lose these funds with a probability of **50%**.

The rest of the questions were opinion-based and therefore had no right or wrong answers.

Next

Start of Round Decision

This is round 1 of 10.

So far the group has contributed £0.00 towards the target of £30.00.

You have £20.00 left in your private account.

How much in £ would you like to transfer to the group account on this round?

○ £0.00 ○ £0.75 ○ £1.50

Next

End of Round Results

Results of round 5 of 10:

After 5 rounds, there is now a total of £15.00 in the group account.

Next

Start of Round Decision

This is round 6 of 10.

So far the group has contributed £15.00 towards the target of £30.00.

You have £6.25 left in your private account.

How much in £ would you like to transfer to the group account on this round?

○ £0.00 ○ £0.75 ○ £1.50

Next
